# Burkholderia cenocepacia utilizes a type VI secretion system for bacterial competition

**DOI:** 10.1002/mbo3.774

**Published:** 2019-01-09

**Authors:** Helena L. Spiewak, Sravanthi Shastri, Lili Zhang, Stephan Schwager, Leo Eberl, Annette C. Vergunst, Mark S. Thomas

**Affiliations:** ^1^ Department of Infection, Immunity and Cardiovascular Disease, The Medical School The University of Sheffield Sheffield UK; ^2^ VBMI, INSERM, Université de Montpellier Nîmes France; ^3^ Department of Plant and Microbial Biology University of Zurich Zurich Switzerland; ^4^ Present address: Northern Genetics Service, The Newcastle upon Tyne Hospitals NHS Foundation Trust, Institute of Genetic Medicine International Centre for Life Newcastle upon Tyne UK; ^5^ Present address: Section of Molecular Biology, Division of Biological Sciences University of California, San Diego La Jolla California; ^6^ Present address: Analytical Chemistry Synthes GmbH Oberdorf BL Switzerland

**Keywords:** antibacterial, bacterial competition, *Burkholderia*, protein secretion, T6SS, type VI secretion system

## Abstract

*Burkholderia cenocepacia* is an opportunistic bacterial pathogen that poses a significant threat to individuals with cystic fibrosis by provoking a strong inflammatory response within the lung. It possesses a type VI secretion system (T6SS), a secretory apparatus that can perforate the cellular membrane of other bacterial species and/or eukaryotic targets, to deliver an arsenal of effector proteins. The *B. cenocepacia* T6SS (T6SS‐1) has been shown to be implicated in virulence in rats and contributes toward actin rearrangements and inflammasome activation in *B. cenocepacia*‐infected macrophages. Here, we present bioinformatics evidence to suggest that T6SS‐1 is the archetype T6SS in the *Burkholderia *genus. We show that *B. cenocepacia *T6SS‐1 is active under normal laboratory growth conditions and displays antibacterial activity against other Gram‐negative bacterial species. Moreover, *B. cenocepacia* T6SS‐1 is not required for virulence in three eukaryotic infection models. Bioinformatics analysis identified several candidate T6SS‐dependent effectors that may play a role in the antibacterial activity of *B. cenocepacia* T6SS‐1. We conclude that *B. cenocepacia* T6SS‐1 plays an important role in bacterial competition for this organism, and probably in all *Burkholderia* species that possess this system, thereby broadening the range of species that utilize the T6SS for this purpose.

## 1 INTRODUCTION

Bacteria utilize many systems to establish a niche, including mechanisms to exploit eukaryotic organisms and/or to compete effectively with other bacterial species colonizing the same ecosystem. Many Gram‐negative bacteria possess a protein secretion system termed the type VI secretion system (T6SS) that participates in one or both processes, depending on the species (Ho, Dong, & Mekalanos, 2014). The T6SS is found in ~25% of Gram‐negative species (Bingle, Bailey, & Pallen, 2008), including the human pathogens *Pseudomonas aeruginosa *(Mougous et al., 2006), *Vibrio cholerae *(Pukatzki et al., 2006), *Serratia marcescens *(Murdoch et al., 2011), and *Burkholderia pseudomallei *(Burtnick et al., 2011). The system is composed of multiple copies of at least thirteen different subunits (TssA‐TssM) and a single copy of the PAAR protein, which are organized into a dynamic protein injection machine containing two distinct interacting subassemblies (Basler, 2015). The first is a contractile structure that shares homology with components of the T4 bacteriophage tail and is comprised of multimers of TssD (also termed Hcp) that assemble into a tube that is sharpened at one end by a trimer of TssI (also known as VgrG) subunits capped by a monomer of the PAAR protein. The tube, in turn, is surrounded by a contractile sheath composed of polymerized TssBC subunits. The tube–sheath structure is assembled on a platform known as the baseplate that consists of the TssEFGK subunits (Brackmann, Nazarov, Wang, & Basler, 2017; Brunet, Zoued, Boyer, Douzi, & Cascales, 2015; Leiman et al., 2009; Nguyen et al., 2017). The second subassembly, composed of the TssJLM subunits, is a channel/chamber complex that spans the inner membrane, periplasm, and outer membrane, and serves to anchor the contractile machinery to the bacterial cell envelope (Brunet et al., 2015; Durand et al., 2015; Nguyen et al., 2017). The role of the TssA subunit is less certain, but it has been proposed to play roles in priming and polymerization of the tube–sheath structure or act as a baseplate component (Planamente et al., 2016; Zoued et al., 2016).

Contraction of the sheath against the baseplate drives the sharpened inner tube through the chamber complex to the exterior where it punctures the cellular membrane of a neighboring target cell. Effector proteins, which may be noncovalently associated with the TssD, TssI, or PAAR subunits (“cargo” effectors) or occur as additional domains on these proteins (“specialized” effectors), are thus delivered into the target cell where they kill or subvert the recipient (Durand, Cambillau, Cascales, & Journet, 2014). In many T6SS‐containing bacteria, these targets are other competing species of bacteria, and so the system plays a major role in bacterial competition (Diniz & Coulthurst, 2015; Hood et al., 2010; MacIntyre, Miyata, Kitaoka, & Pukatzki, 2010; Schwarz et al., 2010). Such T6SS‐dependent competition can occur in a variety of environments, including plant hosts (Ma, Hachani, Lin, Filloux, & Lai, 2014) or the mammalian gut (Chassaing & Cascales, 2018; Sana et al., 2016; Zhao, Caro, Robins, & Mekalanos, 2018). Some T6SSs also specifically target eukaryotic cells and have more of a direct role in virulence, including the T6SS‐5 of *B. pseudomallei* and H2‐ and H3‐T6SS of *P. aeruginosa* (Burtnick et al., 2011; Jiang, Waterfield, Yang, Yang, & Jin, 2014; Sana et al., 2012).

A variety of T6SS‐dependent effectors and cognate immunity proteins have now been described, including superfamilies of antibacterial effectors. These include effectors that target the peptidoglycan layer, phospholipid membrane, or host DNA/RNA, such as the amidase effector–immunity pairs termed Tae‐Tai (for type VI amidase effector/immunity; Hood et al., 2010; Russell et al., 2011; Russell et al., 2012; Fritsch et al., 2013), the type VI lipase effectors (Tle) that possess phospholipase A_1_, A_2_, or D activity (Russell et al., 2013), or the type VI DNase effectors (Tde; Ma et al., 2014), respectively. A number of anti‐eukaryotic effectors have also been described, including a *P. aeruginosa* effector with phospholipase D activity that can target both bacterial and eukaryotic cells (Jiang et al., 2014), the catalase effector, KatN, responsible for intramacrophage survival of enterohemorrhagic *E. coli* (Wan et al., 2017), and a VgrG subunit with a C‐terminal actin cross‐linking domain utilized by *V. cholerae *(VgrG‐1) that impairs the phagocytic activity of eukaryotic host cells (Ma, McAuley, Pukatzki, & Mekalanos, 2009; Pukatzki, Ma, Revel, Sturtevant, & Mekalanos, 2007).

The genus *Burkholderia* constitutes a large and diverse group of Gram‐negative bacterial species, including primary and opportunistic human pathogens, plant pathogens, and plant‐associated species with biocontrol properties (Eberl & Vandamme, 2016). Recently, the classification of the *Burkholderia* has undergone a proposed revision, with all members of the *Burkholderia cepacia* complex (Bcc) and Pseudomallei groups, together with some phytopathogenic species, remaining as *Burkholderia,* while all the other species (typically nonpathogenic environmental strains) have been reassigned to the new genera *Paraburkholderia* (Sawana, Adeolu, & Gupta, 2014) and *Caballeronia *(Dobritsa, Linardopoulou, & Samadpour, 2017). The Bcc is a group of at least twenty closely related species that have gained notoriety as opportunistic respiratory pathogens in cystic fibrosis (CF) patients, as some strains are highly transmissible between individuals and the resulting infections can be difficult to treat effectively and result in fatal pneumonia and septicemia (Depoorter et al., 2016; Drevinek & Mahenthiralingam, 2010). One of the most prevalent Bcc species in CF infections is *B. cenocepacia*. However, despite many studies investigating the virulence mechanisms of this bacterium, the molecular pathogenesis of *B. cenocepacia *infection is not fully understood. Numerous strategies have been proposed to account for its virulence, including its ability to invade and survive intracellularly within host cells (Burns et al., 1996; Cieri, Mayer‐Hamblett, Griffith, & Burns, 2002; Gavrilin et al., 2012; Martin & Mohr, 2000; McKeon, McClean, & Callaghan, 2010; Mesureur et al., 2017), induce pro‐inflammatory responses (Kotrange et al., 2011; Mesureur et al., 2017), scavenge iron (reviewed in Butt & Thomas, 2017), and secrete hydrolytic enzymes such as zinc metalloproteases (Corbett, Burtnick, Kooi, Woods, & Sokol, 2003; Sokol et al., 2003).

As many as eight different T6SSs have been identified across the redefined *Burkholderia* genus, with anywhere up to six of them being encoded in the genome of an individual species (Angus et al., 2014; Shalom, Shaw, & Thomas, 2007). The six T6SSs in *B. pseudomallei* have been described using two numbering systems (Schell et al., 2007; Shalom et al., 2007), with a further two T6SSs identified in other *Burkholderia* species referred to as T6SSa and T6SSb (Angus et al., 2014). In the present investigation, we have adopted the nomenclature of Shalom et al., 2007, and for consistency, we refer to T6SSa and T6SSb as T6SS‐7 and T6SS‐8, respectively. *B. cenocepacia* strains are generally considered to contain only a single T6SS that corresponds to T6SS‐1 of *B. pseudomallei* and *B. thailandensis* (Angus et al., 2014; Aubert, Flannagan, & Valvano, 2008; Aubert, Hu, & Valvano, 2015; Schwarz et al., 2010; Shalom et al., 2007).

The T6SS‐1 in the epidemic *B. cenocepacia* CF isolate K56‐2 was shown to contribute to bacterial survival within a rat model of chronic lung infection (Hunt, Kooi, Sokol, & Valvano, 2004). Subsequent work has suggested that T6SS‐1 is responsible for the ability of *B. cenocepacia* to subvert predatory eukaryotic cells, including the amoeba *Dictyostelium discoideum* and murine and human monocyte‐derived macrophages, and this involves actin cytoskeletal rearrangement (Aubert et al., 2008; Xu et al., 2014). The T6SS‐1 has been shown to exert its effect on cytoskeletal rearrangement through Rho GTPase inactivation (Aubert et al., 2008; Flannagan et al., 2012; Keith, Hynes, Sholdice, & Valvano, 2009; Rosales‐Reyes, Skeldon, Aubert, & Valvano, 2012). More recent studies have suggested that the T6SS‐dependent interactions between *B. cenocepacia* and human‐derived phagocytic cells are important for triggering an innate immune response through pyrin inflammasome activation upon GTPase inactivation, which may promote bacterial clearance and protection from potentially lethal infections in a mouse model (Aubert et al., 2016; Gavrilin et al., 2012; Xu et al., 2014). Several observations which have been attributed to T6SS‐1 activity have been obtained using a *B. cenocepacia* strain in which *atsR*, a gene encoding a hybrid sensor kinase, has been deleted. This results in upregulation of the system and allows for detection of T6SS‐1 secretion activity in a *B. cenocepacia* strain (Aubert et al., 2008, 2015).

Here, we present a bioinformatics analysis of the T6SS‐1 in the genus *Burkholderia* and related species. We demonstrate sufficient T6SS‐1 secretion activity in *B. cenocepacia* isolates growing under standard laboratory conditions to investigate the role of the T6SS in this Bcc species, without the need for upregulation of the system by *atsR* inactivation. From this, we provide evidence to support a functional role of the T6SS‐1 in *B. cenocepacia* in bacterial competition through a series of bacterial competition assays. The contribution of the T6SS‐1 to pathogenesis in three established eukaryotic models of *B. cenocepacia* infection was also investigated, but our results indicated that the system does not contribute to pathogenesis in these models.

## 2 MATERIALS AND METHODS

### 2.1 Strains, plasmids, and growth conditions

The bacterial strains and plasmids used in this study are indicated in Table A1 (Appendix 1). For cultivation of bacteria, strains were routinely grown in LB medium (*E. coli, P. putida*) or M9 minimal salts agar containing 0.5% glucose (*B. cenocepacia*) at 37°C. M9 minimal salts contained 42 mM Na_2_HPO_4_, 22 mM KH_2_PO_4_, 19 mM NH_4_Cl, 9 mM NaCl, 1 mM MgSO_4_, and 0.1 mM CaCl_2_. Antibiotics were used, when appropriate, at the following concentrations: ampicillin (Ap), 100 μg/ml (*E. coli*); chloramphenicol (Cm), 25 μg/ml (*E. coli, P. putida*) and 50–100 μg/ml (*B. cenocepacia*); kanamycin (Km), 50 μg/ml (*E. coli* and *B. cenocepacia*); rifampicin (Rf), 100 μg/ml (*E. coli *and *B. cenocepacia*); and trimethoprim (Tp), 25 μg/mL (*E. coli*), 25 μg/ml (*B. cenocepacia* H111 and Pc715j), and 100 μg/ml (*B. cenocepacia *K56‐2). For selection of trimethoprim resistance in *E. coli*, Iso‐Sensitest Agar (Oxoid) was employed, and for selection of kanamycin resistance in *B. cenocepacia*, Lennox agar was utilized. Dialyzed brain‐heart infusion (D‐BHI) broth was prepared according to Sokol, Ohman, and Iglewski (1979) and used as the liquid growth medium for cultures of *B. cenocepacia *undergoing secreted protein extraction.

### 2.2 DNA preparation and manipulation

Recombinant DNA techniques were performed essentially as described in Sambrook et al. (1989). DNA amplification by PCR was performed with KOD DNA polymerase enzyme (Millipore) or GoTaq G2 Flexi DNA Polymerase (Promega) according to manufacturer's instructions using boiled cell lysate as template DNA. Primers used in this study are indicated in Table A2 (Appendix 1) and were purchased from Eurogentec, Belgium. PCR products were purified from solution or by agarose gel extraction using a QIAquick PCR Purification Kit (Qiagen). DNA restriction enzymes were purchased from Promega or New England Biolabs. DNA was ligated using T4 DNA ligase (Promega). Nucleotide sequence determination was performed by the Core Genomic Facility at The University of Sheffield, UK. Genome sequencing was provided by MicrobesNG (https://www.microbesng.uk), Birmingham, UK. These sequence data have been submitted to the NCBI GenBank database under accession number MK051000. Details of data submission can be found at www.ncbi.nlm.nih.gov/genbank/.

### 2.3 Construction of *B. cenocepacia* strains and plasmids

*Burkholderia* *cenocepacia* chromosomal mutants with insertionally inactivated genes were generated by allelic replacement using the suicide vector pSHAFT2, as previously described (Shastri et al., 2017). Briefly, DNA fragments containing ~1,200 bp of the N‐terminal coding region of *tssM (tssM’)* and the entire *tssK* and *tagY* genes were amplified from *B. cenocepacia* H111 using primer pairs tssMfor and tssMrev, tssKfor and tssKrev, and tagYfor and tagYrev, respectively. Each gene/gene fragment was cloned into the vectors pBBR1MCS or pBluescriptII, where *tssK* was cloned between the restriction sites *Hin*dIII and *Bam*HI, *tssM’* between *Xba*I and *Xho*I, and *tagY *between *Bam*HI and *Xho*I, generating pBBR1‐tssK, pBBR1‐tssM’, and pBluescript‐tagY. To disrupt each target gene, pBBR1‐tssK was restricted with *Eco*RI, pBBR1‐tssM’ with *Bam*HI, and pBluescriptII‐tagY with *Zra*I, and ligated to the trimethoprim (*dfrB2*) resistance cassette that was excised from p34E‐Tp by *Eco*RI, *Bam*HI, and *Sma*I, respectively. The disrupted alleles, *tssK*::Tp, *tssM*::Tp, or *tagY*::Tp, were then transferred to pSHAFT2 as *Xho*I‐*Not*I (*tssK* and *tssM*) or *Xho*I‐*Xba*I (*tagY*) fragments. pSHAFT2‐derived constructs were conjugated into *B. cenocepacia* strains H111, K56‐2, and Pc715j using *E. coli* donor strain S17‐1(λpir) according to Herrero, Lorenzo, and Timmis (1990) and de Lorenzo and Timmis (1994) and selected using M9 agar containing trimethoprim. The previously constructed pSHAFT2‐tssA::Tp plasmid was similarly introduced into K56‐2 and Pc715j. Double crossover recombinants were identified by chloramphenicol sensitivity and verified by PCR using primers pairs that annealed to genomic regions of the target gene located just outside the homologous region contained within the pSHAFT2 construct. See Appendix 2 for further details. Construction of the *B. cenocepacia* H111 *tssM* in‐frame deletion mutant has been described previously (Dix et al., 2018). The *tssM* complementation plasmid, pBBR1‐tssM(+), was constructed by amplifying *tssM* from *B. cenocepacia* H111 with primers tssMforAcc65I and tssMrevXbaI, and ligating the amplicon to the *Acc*65I and *Xba*I sites of pBBR1MCS, which places *tssM* under control of the vector *lacZ* promoter.

### 2.4 Extraction and detection of extracellular proteins

Culture supernatants were collected from 15 ml D‐BHI broth cultures of *B. cenocepacia *strains grown at 37°C until at OD_600_ of 0.6–0.8 and filter sterilized using a 0.22‐μM syringe‐driven filter unit. Sodium deoxycholate was added to supernatants to a final concentration of 0.2 mg/ml, which were then incubated on ice for 30 min. To precipitate proteins, TCA was added at 10% (w/v) final concentration and incubated overnight at −20°C. Supernatants were centrifuged to collect the protein pellets, which were then washed with acetone, collected by centrifugation, and air‐dried. Protein pellets were resolubilized with 15 μl of 1x SDS‐loading buffer (125 mM Tris‐HCl, 5% (w/v) SDS, 10% (v/v) glycerol, 5% (v/v) 2‐mercaptoethanol, 0.005% (w/v) bromophenol blue, pH 6.8). For cell‐associated protein fractions, the whole‐cell pellet was concentrated 20‐fold in PBS and combined with an equal volume of 2x SDS‐sample buffer.

Protein samples were separated in a 15% SDS–polyacrylamide gel, transferred onto 0.45‐μM PVDF membrane (Millipore), and incubated for 1 hr in blocking solution (5% (w/v) milk, TBS, 0.05% (v/v) Tween‐20). TssD secretion was analyzed by Western blotting as standard protocol using a custom rat antibody raised against purified recombinant TssD (The University of Sheffield Biological Services, 1:2,000) and goat anti‐rat HRP secondary antibody (SouthernBiotech, 1:5,000). RNA polymerase β‐subunit was detected as a lysis control using a monoclonal mouse anti‐RNA polymerase β‐subunit primary antibody (1:2,500, NeoClone) and rabbit anti‐mouse HRP secondary antibody (Thermo Scientific, 1:5,000).

### 2.5 Bacterial competition assay

Attacker (*B. cenocepacia*) and prey (e.g., *P. putida*, *E. coli *CC118(λpir)) strains were grown overnight in LB at 37°C. Each culture was then normalized to an OD_600_ of 0.5. Bacterial suspensions were combined in a 5:1 ratio of attacker:prey. Monoculture controls of target and attacker strains with LB were included using the same number of bacteria as in the attacker:prey sample, respectively. 25 μl of each coculture and control culture was spread over a 0.45‐μm nitrocellulose filter membrane on a prewarmed LB agar plate and incubated at 30°C for 4 hr. After incubation, bacteria from each filter membrane were harvested in 1 ml LB and 10^−1^ to 10^−5^ serial dilutions made. 10 μl of each dilution was spotted onto selection plates in triplicate using the surface viable count method (Miles, Misra, & Irwin, 1938). *B. cenocepacia* was selected by Tc resistance, *P. putida* by Cm resistance, *E. coli* CC118(λpir) by Rf resistance, and *E. coli* SM10(λpir) by Km resistance. Plates were incubated at either 37°C or 30°C overnight, dependent on the strain. The number of viable CFU was counted and used to calculate the CFU/mL for each coculture or control culture tested. All experiments were carried out at least three times.

### 2.6 *Galleria mellonella* larvae killing assay

Final‐instar *Galleria mellonella *larvae were purchased fresh from Livefood UK and maintained at 4°C before infection. For preparation of bacteria for injection, *B. cenocepacia *K56‐2 strains were cultured at 37°C in BHI broth until an OD_600_ of 0.6 was reached. The bacteria were centrifuged at 5,000 *g *for 2 min and resuspended in PBS to OD_600_ ~0.5 and serially diluted. For determination of the virulence of the strains, larvae (*n* = 30) were injected with 4 × 10^4^ and 4 × 10^2^ CFU/larvae (in 10 μl) into the hindmost left proleg semi‐automatically using a PB‐600‐1 Repeating Dispenser (Hamilton) affixed to a Gastight 500‐μL Hamilton syringe (Model 1750 RN (large hub) SYR with a 22‐gauge, large hub RN NDL, 2 inch, point style 2 needle). Three control groups (*n* = 20) were injected with 10 μl of sterile PBS, 10 μl heat‐killed bacteria (the lowest dilution of the bacterial culture used for infection boiled at 100°C for 10 min), or left untreated. Serial dilutions of the bacterial suspension were plated onto BHI agar and grown at 37°C overnight to estimate the bacterium inoculum. The heat‐killed bacterial suspension was also spotted onto BHI agar to check sterility. Larvae were incubated at 37°C for 26 hr in sterile plastic Petri dishes lined with filter paper discs. Larval survival was assessed from 16 to 26 hr postinfection at 2‐hr intervals. Dead larvae were classed as those that were stationary and no longer responded to touch. All experiments were carried out at least three times.

### 2.7 *Caenorhabditis elegans* killing assay

Analysis of the virulence of *B. cenocepacia *strains toward *C. elegans* N2 was performed as described in Uehlinger et al. (2009). Briefly, to form a bacterial lawn, overnight cultures of *B. cenocepacia* strains were adjusted to a density of approximately 1.3–1.5 × 10^4^ CFU/ml, and 100 μl of the suspension was plated onto six‐well plates containing nematode growth medium (NGM II) and incubated at 37°C for 24 hr. Following this, approximately 20–40 hypochlorite‐synchronized L4 larvae of *C. elegans* Bristol N2 (obtained from the *Caenorhabditis* Genetics Centre, University of Minnesota, Minneapolis) were used to inoculate the plates. Plates were then incubated at 20°C and the percentage of live worms scored after 48 and 72 hr. Nematodes were considered dead when they failed to respond to touch. *E. coli *OP50 was used as a negative control. All experiments were carried out at least three times.

### 2.8 Zebrafish embryo infection assay

Infection of zebrafish (*Danio rerio*) embryos was performed as described in Vergunst, Meijer, Renshaw, and O’Callagha (2010), Mesureur and Vergunst (2014). Briefly, *B. cenocepacia* K56‐2 and the otherwise isogenic *tssM*::Tp and *tssA*:Tp mutants were grown overnight in LB containing the appropriate antibiotics. Thirty hours postfertilization, zebrafish embryos were dechorionated and anesthetized in E3 medium with 0.02% buffered tricaine methanesulfonate (MS222). Embryos (*n* = 20) were then microinjected with around 100 CFU of bacteria directly into the blood circulation and maintained in E3 medium at 28°C. Embryo survival was monitored at regular intervals from 40 hr postinfection (hpi). Dead embryos were scored as those without a heartbeat. The experiment was carried out twice.

From the same experiments, five infected embryos per treatment group were taken randomly at 0 and 24 hpi and subjected to bacterial enumeration as described in Mesureur & Vergunst, 2014. Statistical analysis was performed using Prism 6 (GraphPad). Survival assays are represented in Kaplan–Meier graphs and analyzed with a log‐rank (Mantel–Cox) test. In CFU count experiments, significance was determined using one‐way ANOVA, with Sidak's multiple comparison test.

### 2.9 Bioinformatic analysis

Relevant DNA and protein sequences were obtained from the NCBI GenBank database (Clark, Karsch‐Mizrachi, Lipman, Ostell, & Sayers, 2016). Unannotated GenBank entries were manually interrogated for coding regions and the respective protein sequences using SnapGene^®^ software (from GSL Biotech; available at http://www.snapgene.com). All protein homology analyses were performed using NCBI blastp and the nonredundant protein sequences (nr) database. T6SS‐1 clusters were identified in a two‐step process. First, the amino acid sequences of TssH (BCAL0347) and TagX proteins (BCAL0352) from *B. cenocepacia* J2315 were used as search queries to identify homologous proteins. Second, the loci encoding these proteins were interrogated for the presence of other T6SS‐related genes. If homologues of the additional *tag* genes *tagM, tagN, *and *tagY *and the majority of core *tss *genes were present, the region was defined as a T6SS‐1 cluster. To identify T6SS‐7 clusters, the protein sequence of the TssH homologue in the H111 T6SS‐7 cluster (I35_RS17330) was used as the query sequence to identify homologous proteins with a percentage sequence identity ≥70% in *Burkholderia* and *Paraburkholderia* species. The surrounding loci were then interrogated. If homologues of the core *tss *genes (*tssA*‐*tssM*) were present in a similar genetic arrangement as that in the H111 T6SS‐7 cluster, the region was defined as a T6SS‐7 cluster.

Multiple sequence alignments were performed using Clustal W or Clustal Omega (Larkin et al., 2007; Sievers et al., 2011) and formatted for display using BoxShade (https://www.ch.embnet.org/software/BOX_form.html). The prediction of transmembrane helices within proteins was performed using TMHMM Server v.2.0 (Krogh, Larsson, Heijne, & Sonnhammer, 2001).

## 3 RESULTS

### 3.1 Comparative analysis of the T6SS‐1 in *Burkholderia* and non‐*Burkholderia* species

**Figure 1:**
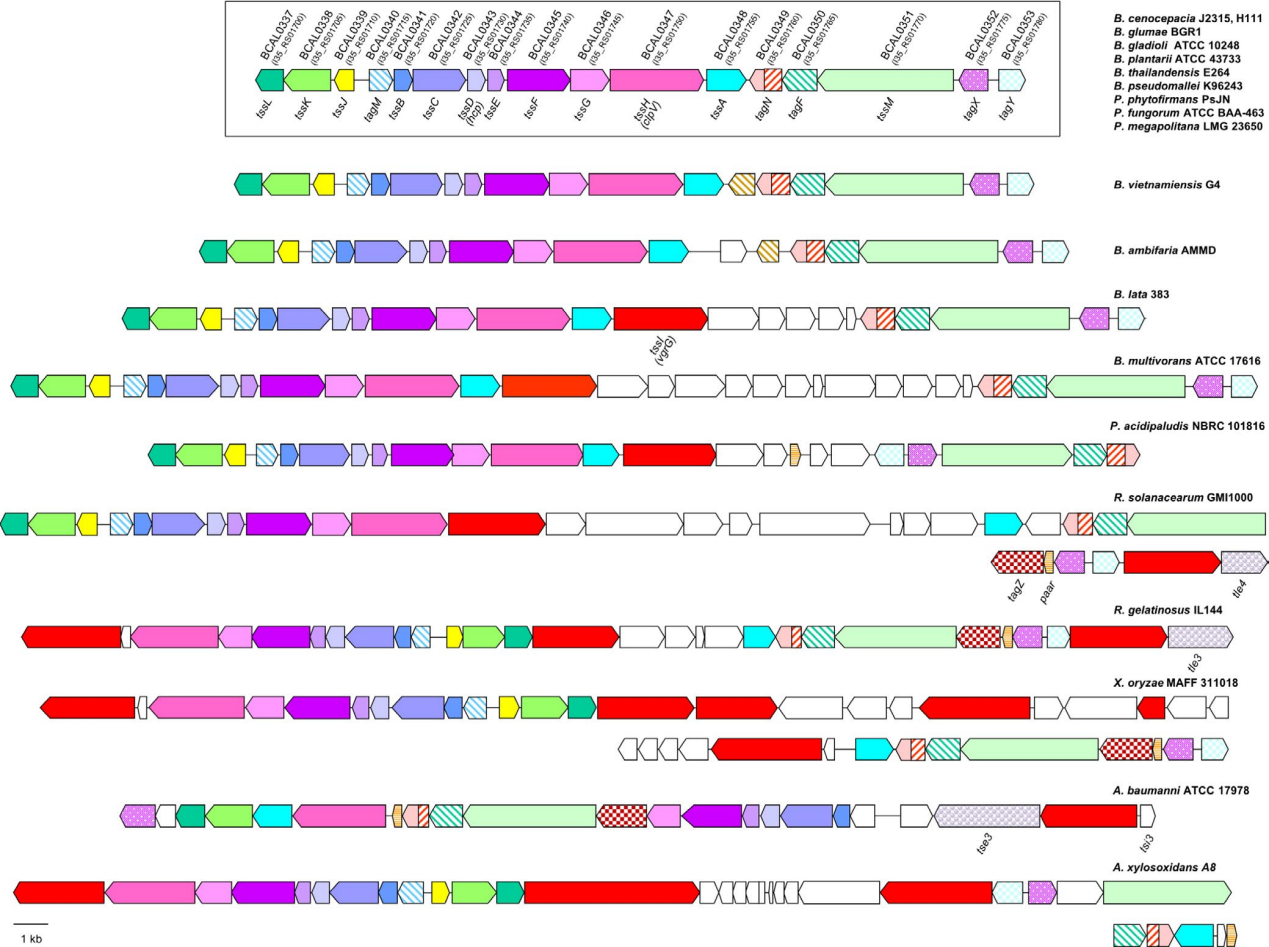
Gene arrangement and distribution of the Burkholderia T6SS‐1 gene cluster. Schematic representation of the *Burkholderia* T6SS‐1 gene cluster and related gene clusters in members of the *Proteobacteriaceae*. The box shows the genetic organization of the archetype *Burkholderia* T6SS‐1 gene cluster harbored by the indicated species, including *B. cenocepacia* (for reference, the *B. cenocepacia* T6SS‐1 gene cluster corresponds to BCAL0337‐BCAL0353 in strain J2315 and I35_RS01700‐I35_RS01780 in H111, as indicated). Variations on the same basic theme found in other members of the *Burkholderia*, related genera within the β‐proteobacteria (*Achromobacter*, *Paraburkholderia*, *Ralstonia*, and *Rubrivivax*), and some members of the γ‐proteobacteria (*Acinetobacter*, *Xanthomonas*) are shown

In a previous study, six T6SSs were identified in *B. pseudomallei *(Shalom et al., 2007). The only one encoded on the large chromosome (T6SS‐1) has been identified in nine other *Burkholderia* species and three members of the *Paraburkholderia* (Angus et al., 2014). We have extended this analysis to include all *Burkholderia *and *Paraburkholderia *species, and members of other related proteobacteria for which genome sequence information is available. Therefore, the amino acid sequence of protein products encoded by the T6SS‐1 gene cluster of *B. cenocepacia* J2315 was used in blastp searches to identify homologous proteins in other *Burkholderia*, *Paraburkholderia, *and related species, and the respective T6SS‐1 gene clusters that encoded them were identified. All members of the genus *Burkholderia* (i.e., the Bcc and pseudomallei groups and the phytopathogenic strains *B. gladioli*, *B. plantarii*, and *B. glumae*), with the exception of the recently described species *B. singularis,* were found to harbor the T6SS‐1 gene cluster (Table A3 in Appendix 1). In species for which a complete genome assembly was available, the T6SS‐1 was located on chromosome 1 in every case. We also found homologous loci of the *Burkholderia *T6SS‐1 gene cluster in many species of the closely related *Paraburkholderia* genus, including *P. acidipaludis*, *P. phytofirmans*, and *P. fungorum *(see Table A4 in Appendix 1 for additional species), several of which were located on chromosome 2 or 3 instead of chromosome 1. The T6SS‐1 cluster of *P. acidipaludis* is shown in Figure 1. A T6SS‐1 cluster with a similar, but not identical, genetic organization was also found in other β‐proteobacteria, including *Ralstonia solanacearum*, *Rubrivivax gelatinosus*, *Achromobacter xylosoxidans*, and the *γ*‐proteobacteria species *Xanthomonas oryzae* and *Acinetobacter baumannii *(Figure 1)*.*

Most clusters were found to contain the core *tss* genes on three closely linked transcriptional units. However, in the majority of Bcc species, genes encoding the core T6SS subunits TssI and PAAR were not observed to be located in the T6SS‐1 gene cluster and are instead present in multiple copies at other loci distributed throughout the genome (as observed for *B. cenocepacia *by Aubert et al., 2015). Curiously, the T6SS‐1 gene cluster of members of the genus *Acinetobacter* lacked a copy of the core *tssJ* gene, as previously noted (Weber et al., 2013). It was also observed that several T6SS‐1 clusters contained insertions of one or more additional genes between the core genes or translocations of gene blocks, such as those in *B. multivorans*, *P. acidipaludis*, and *R. solanacearum* (Figure 1).

Type VI‐associated genes (*tag*) are conserved in some T6SSs but not others and encode proteins related to T6SS function, such as regulators or auxiliary subunits (Aschtgen, Thomas, & Cascales, 2010; Lossi et al., 2012; Shalom et al., 2007; Silverman et al., 2011). Five *tag* genes were recognized as being conserved in almost all T6SS‐1 clusters. These are *tagF*, which encodes a post‐translational regulator and is also present in some unrelated T6SSs such as the H1‐T6SS of *P. aeruginosa* (Lin et al., 2018; Silverman et al., 2011); *tagM*, encoding a putative outer membrane‐anchored lipoprotein of unknown function (Shalom et al., 2007); *tagN*, encoding a putative PG‐anchoring protein (Aschtgen et al., 2010); *tagX*, encoding a Sec‐dependent membrane‐anchored peptidoglycan hydrolase that facilitates T6SS sheath assembly through formation of holes in the peptidoglycan layer (Aubert et al., 2015; Ringel, Hu, & Basler, 2017; Weber et al., 2016); and a previously undescribed gene referred to here as *tagY*.

*tagY* corresponds to BCAL0353 in *B. cenocepacia* J2315 and is located upstream from *tagX*, but in the reverse orientation in nearly all T6SS‐1 gene clusters (Figure 1). It does not occur in unrelated T6SS gene clusters. In most *Burkholderia* species, *tagY* is likely to constitute a monocistronic operon due to the presence of a putative Rho‐independent transcription termination sequence located downstream from the *tagY* coding sequence, but in some non‐*Burkholderia* species, it constitutes the first gene of a polycistronic operon that encodes additional T6SS‐related proteins such as TssI and putative Tle effectors (Figure 1). Therefore, TagY is likely to play a role in the activity of T6SS‐1. It should be noted that *tagM* and *tagY* are not present in the *Acinetobacter* T6SS‐1 gene cluster. As members of this genus also appear to lack a TssJ orthologue, they are devoid of three periplasmic proteins that are present in the *Burkholderia*‐type T6SS‐1 in other species.

**Figure A1:**
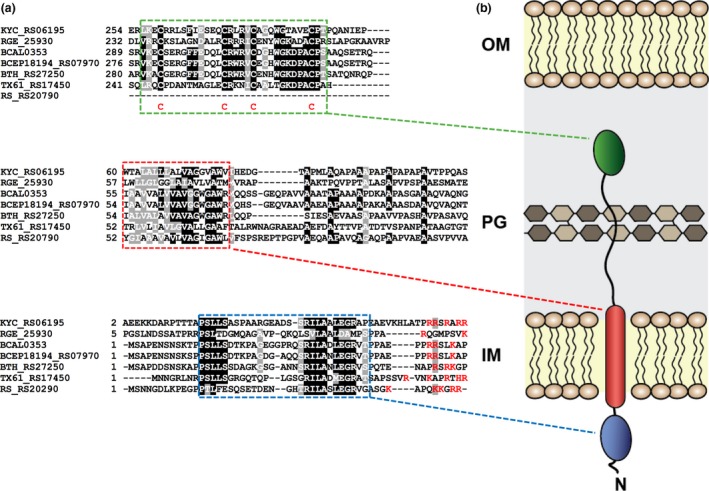
*tagY *encodes a predicted transmembrane protein with a periplasmically located C‐terminal domain containing four cysteine residues. (A) Amino acid sequence alignment of TagY homologues. The amino acid sequences of TagY homologues identified in *B. cenocepacia* J2315 (BCAL0353), *A. arsenitoxydans *SY8 (KYC_RS0619), *R. gelatinosus* IL144 (RGE_25930), *B. lata* 383 (BCEP18194_RS07970), *B. thailandensis *E264 (BTH_RS27250), *X. oryzae* MAFF 311018 (TX61_RS17450) and *R. solanacearum* GMI1000 (RS_RS20790) were aligned using Clustal W. Note that the homologue in *R. solanacearum *does not contain the cysteine rich C ‐terminal domain. Sequences constituting the predicted N‐terminal domain (NTD), transmembrane domain (TMD), and cysteine‐rich periplasmic C‐terminal domain (CTD) are indicated by blue, red and green boxes, respectively. Positively charged amino acids located N‐terminal to the TMD are shown in red font. (B) Schematic representation of the predicted domain arrangement of TagY*. *The small cytoplasmic NTD (blue), TMD (red), long linker region (black) and cysteine‐rich periplasmic CTD (green) are shown.

Analysis of the predicted protein product of *tagY *orthologues identified a putative transmembrane domain (TMD) located approximately 55 residues from the N‐terminus. The region located N‐terminal to the TMD contains two short conserved motifs separated by 10–13 amino acids (Appendix Figure A1). Based on the “positive inside rule” (Elofsson & von Heijne, 2007), the presence of amino acid residues with basic side chains immediately N‐terminal to the TMD suggests that the N‐terminal region constitutes a small cytoplasmically located domain. The TMD is followed by a long linker‐like region of low complexity, which in TagY orthologues in the *Burkholderia* spp. shares homology to the RnfC barrel sandwich hybrid domain (cl26195), a domain found at the N‐terminus of the RnfC electron transport complex subunit in *Rhodobacter capsulatus *(Biegel, Schmidt, González, & Müller, 2011; Schmehl et al.., 1993). A conserved C‐terminal region of ~40 amino acids that contain four cysteine residues was identified in most TagY orthologues (Appendix Figure A1). Due to the known role of cysteine thiols in various cellular activities, it is possible that this part of the protein, which is predicted to be located in the periplasmic space, constitutes a domain which assembles an iron–sulfur cluster. Alternatively, it may be involved in binding other transition metal ions such as zinc or copper, or act as a redox sensor.

**Figure A2:**
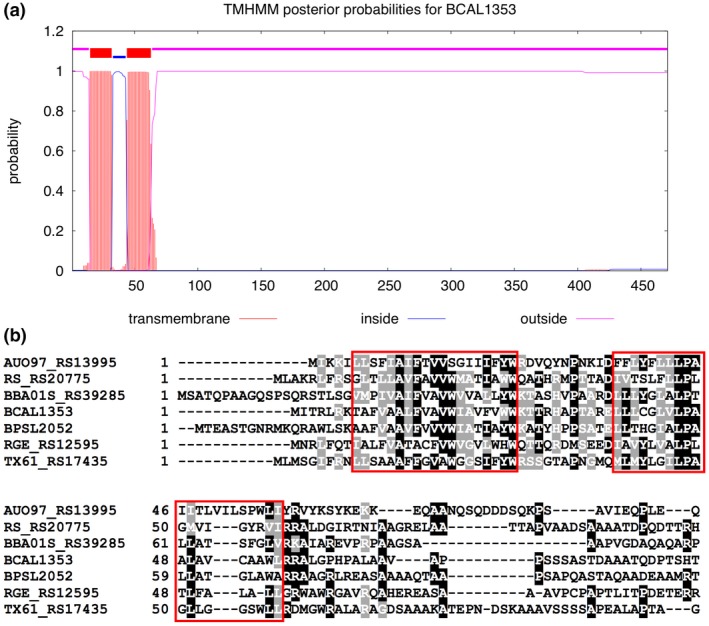
*tagZ* encodes a putative cytoplasmic membrane protein. (A) Schematic representation of the predicted topology and transmembrane helices within TagZ of *B. cenocepacia* J2315 (encoded by BCAL1353), as predicted by TMHMM Server (v. 2.0). (B) N‐terminal amino acid sequence alignment of TagZ homologues in *B. cenocepacia* J2315 (BCAL1353), *B. pseudomallei* K96243 (BPSL2052), *R. gelatinosus* IL144 (RGE_RS12595), *X. oryzae* MAFF 311018 (TX61_RS17434), *P. bannensis* NBRC 103871 (BBA01S_RS39285), *R. solanacearum* GMI1000 (RS_RS20775) and *A. baumannii* ATCC 17978 (AUO97_RS13995). The predicted transmembrane helices are enclosed in red boxes.

**Figure A3:**
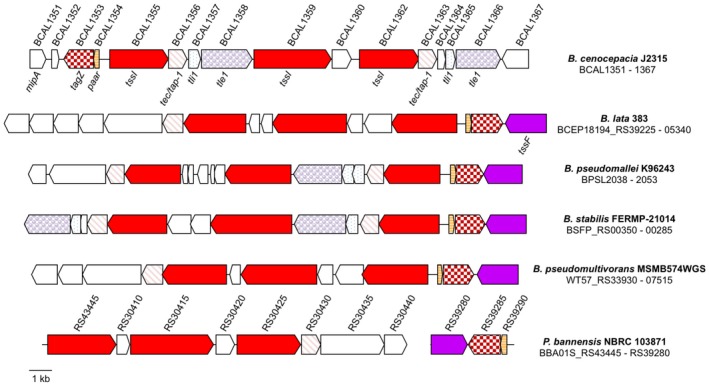
*tagZ* and *paar* genes associated with the *Burkholderia* T6SS‐1 are often located within a triple *tssI* cluster.Schematic representation of triple *tssI* gene clusters identified in the indicated *Burkholderia* spp. and *Paraburkholderia bannensis* NBRC 103871, which contain the T6SS‐1‐associated *tagZ* and *paar* genes. Genes encoding putative T6SS‐lipase effectors (*tle1*), associated lipase immunity protein(s) (*tli1*) and T6SS adaptor proteins (*tec/tap‐1*) are often present within the cluster.

Two additional genes are conserved in the T6SS‐1 cluster of species that are not members of the *Burkholderia* and *Paraburkholderia* genera. They correspond to RSp0764 and RSp0765 of *R. solanacearum* GM1000, RGE_RS12595 and RGE_RS12600 of *R. gelatinosus* IL144, XOO3320 and XOO3321 of *X. oryzae* MAFF 311018, AT699_RS16195, and an unannotated gene of *A. arsenitoxydans* NCTC10807, and ABAYE2409 and ABAYE2405 of *A. baumannii* (which were previously annotated as *asaB *and *asaC* as they were thought to be unique to the *Acinetobacter* T6SS; Carruthers, Nicholson, Tracy, & Munson, 2013). Bioinformatic analysis predicts that the first of each pair of genes encodes a protein possessing TMDs close to the N‐terminus (Appendix Figure A2), whereas the latter has been recognized as a putative PAAR domain‐containing protein in *A. baylyi* and named accordingly (Weber et al., 2016). Homologues of the *asaB* gene (from herein referred to as *tagZ*) are also present in some, but not all *Burkholderia *and in a single *Paraburkholderia* species (*P. bannensis*), while *paar* is present in all *Burkholderia* and *Paraburkholderia* species. However, both genes reside outside the T6SS‐1 cluster in these two genera and in some cases are located within a conserved gene cluster on chromosome 1 that encodes three TssI subunits and one or more effector–immunity protein pairs (Appendix Figure A3). The gene encoding the PAAR domain protein is located immediately upstream of *tagZ* in these T6SS‐related gene clusters, as is the case where these genes occur in the main T6SS‐1 gene cluster (Figure 1). As a number of the *Burkholderia* and *Paraburkholderia* species possess only T6SS‐1, it can be concluded that despite its location outside of the main T6SS‐1 gene cluster, the products of the *paar*‐*tagZ* gene pair play a role in the activity of T6SS‐1.

### 3.2 Identification of an additional, isolate‐specific, type VI secretion system in *Burkholderia cenocepacia*

**Figure 2:**
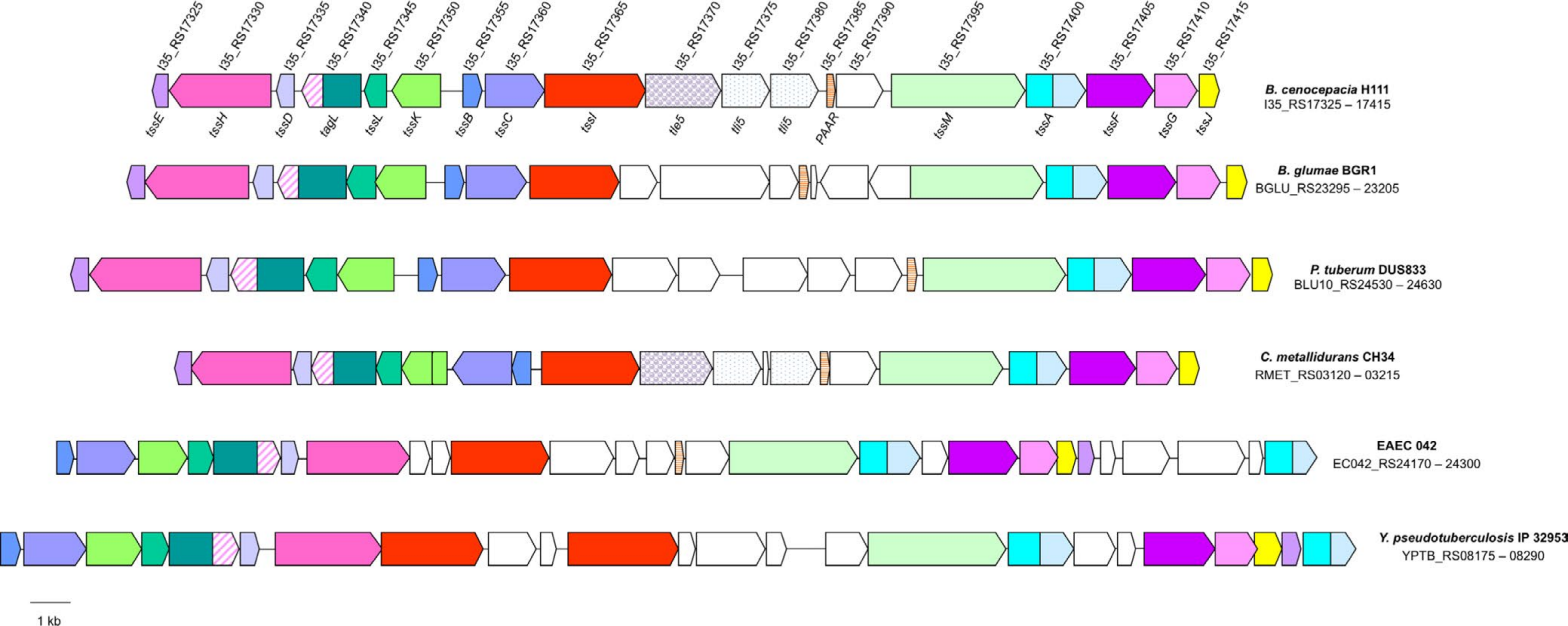
* Burkholderia cenocepacia* H111 possesses an additional T6SS that is present in some plant‐associated and human pathogenic bacteria. Schematic representation of a T6SS gene cluster identified in *B. cenocepacia *H111 (top) (I35_RS17325–I35_RS17415), which has a similar genetic organization to the T6SS‐7 cluster (also known as T6SS‐a) previously identified in *B. glumae* BGR1 and *P. tuberum* DUS833. A related T6SS cluster is also present in *C. metallidurans *CH34, EAEC 042 (the T6SS‐1 or *sci‐1* cluster), and *Y. pseudotuberculosis* IP 32953 (T6SS‐2)

During the bioinformatic analysis of the T6SS‐1 described above, an additional, complete T6SS gene cluster was identified in *B. cenocepacia *strain H111, a cystic fibrosis isolate (Carlier et al., 2014; Geisenberger et al., 2000). Further genome mining revealed that it was also present in *B. cenocepacia* strains FL‐5‐3‐30‐S1‐D7, VC12308, and DWS 37E‐2, and several additional *B. cenocepacia* isolates for which only contig or scaffold‐level genomic data are currently available, including D2AES, PC148, and TAtl‐371 (see Table A5 in Appendix 1 for loci). This second T6SS cluster is located on chromosome 2 in the completely sequenced strains and encodes orthologues of all the core T6SS subunits, including TssI and PAAR (Figure 2). The cluster shares a genetic arrangement that is similar to a T6SS cluster present in the plant pathogenic *Burkholderia* species, *B. glumae*, and to a T6SS gene cluster present in several *Paraburkholderia* species, including *P. tuberum*, which has been referred to as T6SSa (Angus et al., 2014), but for consistency with the established nomenclature is referred to here as the *Burkholderia* T6SS‐7. Our analysis also identified T6SS‐7 clusters in some but not all isolates of other Bcc species and in *Cupriavidus metallidurans*, a species that is closely related to the *Burkholderia*/*Paraburkholderia* clade (Table A5 in Appendix 1 and Figure 2).

*Burkholderia* T6SS‐7 is notable in possessing a TagL orthologue which serves as an auxiliary subunit that anchors the T6SS to the peptidoglycan (Aschtgen et al., 2010). Accordingly, the genetic organization of this T6SS gene cluster is also similar to those encoding TagL‐dependent T6SSs present in human pathogens such as T6SS‐2 of *Yersinia pseudotuberculosis*, the T6SS of the uropathogenic *E. coli* strain CFT073, and the T6SS‐1 (Sci‐1 T6SS) of enteroaggregative *E. coli *(Figure 2).

**Figure A4:**
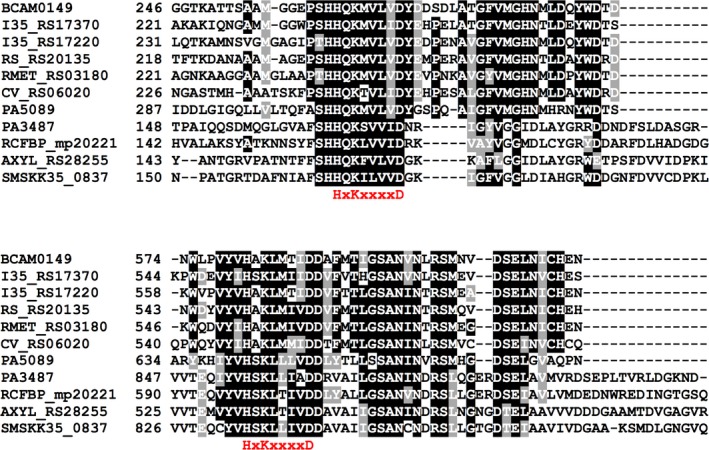
The T6SS‐7 cluster of *B. cenocepacia* H111 encodes a putative T6SS type‐5 lipase effector (Tle5) with characteristic dual HxKhhhhD sequence motifs.Amino acid sequence alignment of the two regions containing HxKhhhhD catalytic motifs present in putative T6SS‐dependent type‐5 lipase effector (Tle5) homologues from *B. cenocepacia* H111 (encoded within the T6SS‐7 cluster at I35_RS17370), *B. cenocepacia* J2315 (BCAM0149), *R. solanacearum* GMI1000 and CFBP2957 (RS_RS20135 and RCFBP_mp20221), *C. violaceum* ATCC 12472 (CV_RS06020), *C. metallidurans* CH42 (RMET_RS03180), *P. aeruginosa* PAO1 (PA5089 and PA3487), *A. xylosoxidans* A8 (AXYL_RS28255) and *S. maltophilia* SKK35 (SMSKK35_0837). The dual HxKhhhhD motif (where h is an amino acid with a hydrophobic side chain) is characteristic of phospholipase D (PLD) enzymes.

Bioinformatic analysis of the T6SS‐7 gene cluster also suggests that it encodes a phospholipase D (PLD) effector and two corresponding Tli immunity proteins in members of the *Burkholderiaceae*. This particular PLD belongs to the Tle5 group of phospholipase effectors and is closely related to the PldB protein, PA5089, encoded by the H3‐T6SS of *P. aeruginosa* that has been shown to serve as a transkingdom effector (Russell et al., 2013; Jiang et al., 2014; Appendix Figure A4).

### 3.3 The *Burkholderia cenocepacia* T6SS‐1 is functional during growth under standard laboratory conditions

The presence of the core T6SS subunit, TssD, in bacterial culture supernatants is the hallmark of an active T6SS and can be used as a method to determine functionality of the T6SS. This assay was used to determine whether *B. cenocepacia* isolates possess an active T6SS‐1 during growth under standard laboratory conditions and to validate T6SS‐1 mutants prior to their use in bacterial competition and virulence assays described below. Therefore, mutants defective in the core *tssA*, *tssK,* and *tssM *components of T6SS‐1 were generated in strains H111, K56‐2, and Pc715j, and TssD secretion of the mutants was compared to that of the corresponding wild‐type parent strains grown in broth culture.

**Figure 3:**
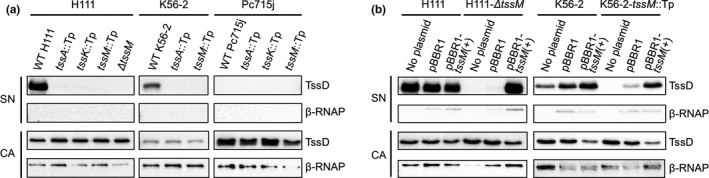
* Burkholderia cenocepacia* T6SS‐1 is active under standard laboratory conditions. Secretion activity of *B. cenocepacia* T6SS‐1 in vitro. Anti‐TssD immunoblot was performed on proteins extracted from culture supernatants (SN) and cell‐associated proteins (CA) of *B. cenocepacia *wild‐type (WT) strains H111, K56‐2, and Pc715j, and corresponding T6SS‐1 mutants (*tssA*::Tp, *tssK*::Tp, *tssM*::Tp, and/or Δ*tssM*) (a) and the H111 and K56‐2 WT and Δ*tssM* or *tssM*::Tp strains carrying a complementation or empty control plasmid (pBBR1‐tssM(+) and pBBR1MCS (“pBBR1”), respectively) (b). Anti‐β‐RNAP antibody was used as an indicator of bacterial cell lysis in preparations. Scales and labels as indicated. The H111 *tssA*::Tp mutant was included as a control

Western blotting showed that TssD was absent in the culture supernatant of the *tssA*, *tssK*, and *tssM *mutants but present in the respective wild‐type H111 and K56‐2 supernatants consistent with previous results obtained using a *B. cenocepacia atsR* mutant (Aubert et al., 2015; Figure 3a). The H111 and K56‐2 *tssM* mutants were subjected to a complementation analysis, whereby TssD secretion could be restored in both strains by introduction of a plasmid expressing *tssM* (Figure 3b). Together, these results indicate that *B. cenocepacia* isolates H111 and K56‐2 have an active T6SS‐1 under standard laboratory conditions.

The additional *B. cenocepacia* isolate analyzed, Pc715j (and its T6SS‐deficient derivatives), was unable to secrete TssD into the extracellular milieu, despite detection of this protein in whole‐cell extracts (Figure 3a), indicating that TssD was being expressed but that the T6SS‐1 was incapable of firing and/or assembly in this strain. Whole‐genome sequencing of our laboratory stock of Pc715j indicated that an IS element was inserted into the *tssM* gene*. *The IS element exhibited homology to the ISUmu23 insertion sequence found in the Bcc‐specific phage KS5 (Lynch, Stothard, & Dennis, 2010), and its insertion into the *tssM* coding sequence was predicted to result in production of a nonfunctional truncated form of the TssM subunit that lacked the C‐terminal 447 amino acids.

The role of the candidate post‐translational regulatory protein, TagY, in T6SS‐1 activity was also explored by inactivating the *tagY* gene in strain H111. However, no significant difference in TssD secretion was observed between the wild‐type and the mutant strains (results not shown). These results could be explained if TagY acts to further upregulate the system in response to an unknown signal that is not present under the assay conditions.

### 3.4 *Burkholderia cenocepacia* T6SS‐1 exhibits antibacterial activity

It has been demonstrated that the T6SS can target effector proteins to other bacteria, thereby helping the organism to compete more effectively against other bacterial species in its growth environment. However, to date, the antibacterial nature of the T6SS‐1 in any member of the *Bcc* has not been reported. Therefore, we addressed the role of the T6SS‐1 in the ability of *B. cenocepacia* to compete effectively with other bacterial species.

As basal‐level TssD secretion appeared to be greater in *B. cenocepacia *H111 than in strain K56‐2 (Figure 3a), the former was chosen to evaluate the role of the T6SS‐1 in competition in this species. Strains H111 and H111‐Δ*tssM* were used as “attackers” in a bacterial competition experiment against Gram‐negative “prey” species *Pseudomonas putida* KT2440, *Escherichia coli* CC118(λpir), and *E. coli* SM10(λpir). Following cocultivation for four hours on solid medium, viable prey bacteria were enumerated and the number that survived attack by the wild‐type and mutant attackers were compared.

**Figure 4:**
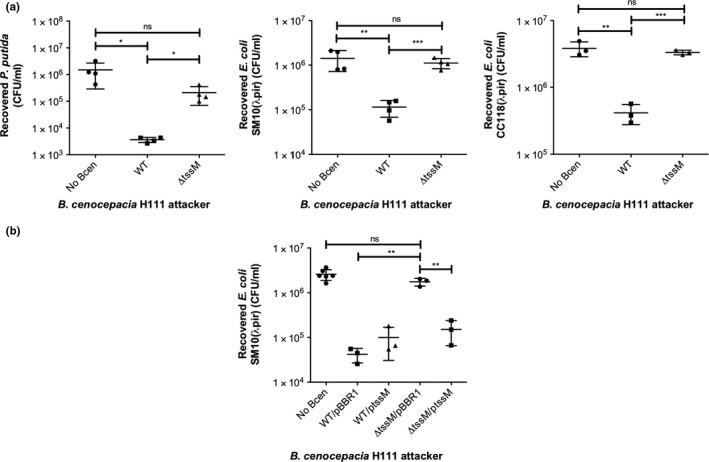
The *Burkholderia cenocepacia* T6SS‐1 plays a role in bacterial competition. (a) Recovery of viable *P. putida*, *E. coli* SM10(λpir) and *E. coli* CC118(λpir) (in CFU/ml) “prey” strains following coculture with the indicated *B. cenocepacia* H111 “attacker” strains for 4 hr at 30°C. (b) Comparison of recovery of *E. coli *SM10(λpir) prey following coculture with *B. cenocepacia* H111 WT or Δ*tssM* mutant attacker strains carrying complementation or control plasmids pBBR1‐tssM(+) (“ptssM”) and pBBR1MCS (“pBBR1”), respectively. n ≥3 and error bars indicate *SD*

**Figure A5:**
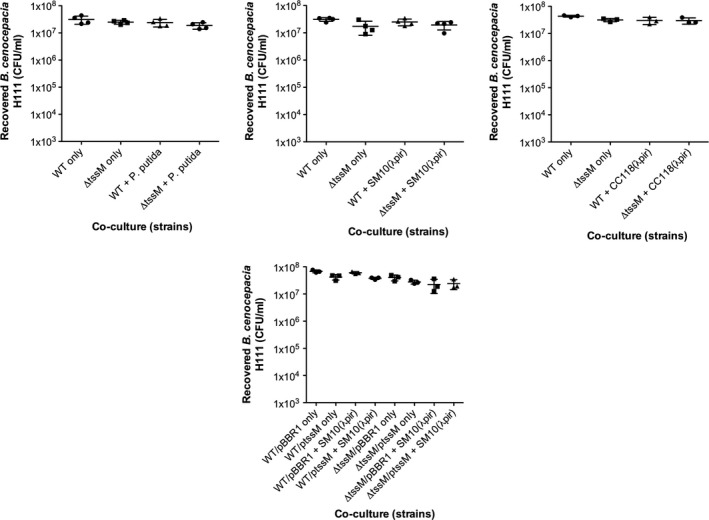
Enumeration of viable *B. cenocepacia* attacker strains in bacterial competition assay.(A) Recovery of viable *B. cenocepacia* H111 ‘attacker’ strains following co‐culture with the indicated prey strains or medium control for 4 hours at 30°C. (B) Comparison of recovery of *B. cenocepacia* H111 WT or Δ*tssM* mutant attacker strains carrying a complementation or control plasmid (pBBR1‐tssM (ptssM) and pBBR1) following co‐culture with *E. coli* SM10(λpir) prey or medium control. *n* ≥ 3 and error bars indicate SD.

For all three prey strains tested, the number of recovered surviving prey bacteria was significantly lower (by one to two orders of magnitude) when they were cocultured with the wild‐type attacker strain in comparison with no attacker, demonstrating that *B. cenocepacia* can restrict the growth of *E. coli* and *P. putida* (Figure 4a). Furthermore, following coculture with the Δ*tssM* attacker strain, the number of surviving prey bacteria was similar to those observed when no attacker was present (Figure 4a). The number of recoverable attacking H111 bacteria was similar for both the WT and T6SS mutant strains and was unaffected by coculture with all prey strains (Appendix Figure A5). To validate these results, a complementation experiment was performed using the *E. coli* SM10(λpir) strain as the prey. The antibacterial activity of the *tssM* mutant attacker toward the *E. coli* strain could be restored to wild‐type levels by introduction of a plasmid expressing *tssM* into the mutant attacker strain (Figure 4b). These data strongly suggest that the T6SS‐1 in *B. cenocepacia *has antibacterial properties.

### 3.5 *Burkholderia cenocepacia* T6SS‐1 is not required for virulence in eukaryotic models of infection

**Figure 5:**
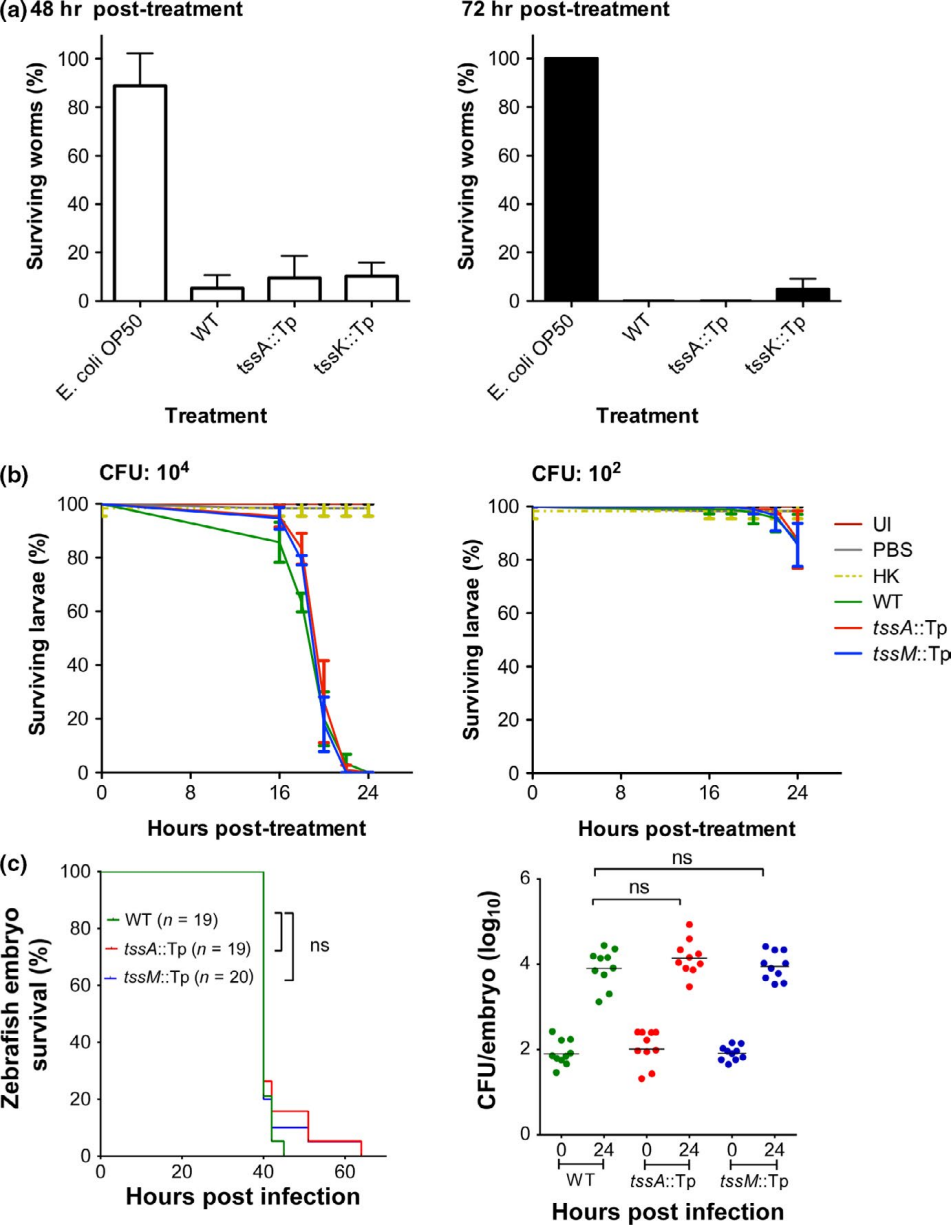
The *Burkholderia cenocepacia* T6SS‐1 is not required for virulence toward eukaryotes. (a) Percentage survival of *C. elegans* following 48‐hr (white bars, left) and 72‐hr (black bars, right) infection with the indicated *B. cenocepacia* H111 strains at 20°C. Twenty to 40 worms were used per condition. *E. coli* OP50 was used as a negative control. Each point indicates mean (*n* = 3), and error bars indicate *SD*. (b) Percentage survival of wax moth larvae following 24‐hr infection with high (1 × 10^4^) (left) and low (1 × 10^2^) (right) doses of *B. cenocepacia* K56‐2 (WT) and indicated mutant strains at 37°C. Thirty larvae were infected per condition. Uninfected (UI), heat‐killed *B. cenocepacia* WT (HK), and mock‐infected (PBS) controls were included. Each point indicates mean % survival (*n* = 3), and error bars indicate *SD*. (c) Zebrafish embryos were microinjected with ~100 CFU of indicated *B. cenocepacia* K56‐2 strains and kept at 28°C in individual wells containing E3 medium. About 20 embryos were used for determination of survival percentage over time (representative experiment shown on the left), and five embryos per indicated time point were used to determine recovery of viable *B. cenocepacia* K56‐2 counts (*n* = 5 per time point per experiment, geometric mean; right‐hand graph, showing summary of two independent experiments). ns: not significant

Several eukaryotic infection models have been used to identify virulence factors of *B. cenocepacia, *including the nematode *C. elegans*, larvae of the waxmoth *G. mellonella*, and zebrafish embryos (Seed & Dennis, 2008; Uehlinger et al., 2009; Vergunst et al., 2010). To ascertain the contribution of T6SS‐1 to the virulence of *B. cenocepacia,* we utilized all three of these infection models. Comparison of the survival of *C. elegans* infected with *B. cenocepacia* strain H111 and its *tssA* and *tssK* mutant derivatives for 48 and 72 hr showed that the wild‐type and mutant strains exhibited a similar killing efficiency during both time periods (Figure 5a). *tssA* and *tssM* mutants of strain K56‐2 were used to explore the role of T6SS‐1 in virulence toward *G. mellonella* larvae and zebrafish embryos. Comparison of the survival of *G. mellonella* following infection with these mutants demonstrated that they were able to kill the larvae as effectively as the wild‐type strain at 24 hr postinfection, whether high or low bacterial loads were employed (4 × 10^4^ and 4 × 10^2^ CFU/larvae, respectively), (Figure 5b). Wild‐type K56‐2 and its T6SS‐1 mutant derivatives were also found to be similarly virulent in the zebrafish model, both in terms of mortality and multiplication of the bacteria in the host (Figure 5c). Taken together, these results suggest that the T6SS‐1 in *B. cenocepacia* is primarily used to target other bacterial species. Although T6SS‐1 does not significantly contribute to virulence in the eukaryotic models tested here, it is possible that it may have an impact in other systems.

### 3.6 In silico identification of putative T6SS‐dependent effectors in *B. cenocepacia*

**Figure A6:**
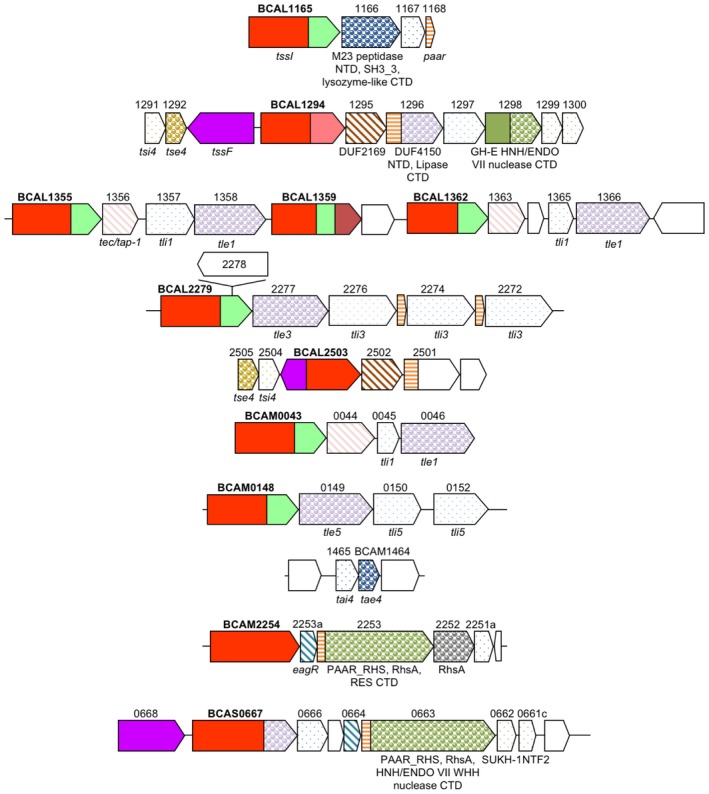
T6SS‐1 effector loci in the *B. cenocepacia* J2315 genome.Schematic representation of *tssI* gene clusters identified in *B. cenocepacia* J2315 and putative T6SS‐dependent effector‐immunity pairs encoded within the clusters (*tssI* genes are shown in red with C‐terminal coding sequences coloured green (DUF2345 domain), brown (metalloprotease domain) or with spheres (lipase_3 domain) as observed previously (Aubert et al., 2015). The BCAL2503 *tssI* gene contains a deletion that results in its fusion to a divergently orientated *tssF* gene and is presumably non‐functional. Genes encoding the putative effectors and associated immunity‐protein are labelled and filled‐in with coloured spheres and dots, respectively. Genes encoding recognized T6SS‐adaptor proteins, Tec/Tap‐1, DUF2169‐encoding proteins and EagR, and PAAR‐containing proteins are also labelled and filled with diagonal coloured stripes and orange horizontal stripes, respectively. *tssF* homologues are also shown (purple). The BCAM1464‐BCAM1465 *tae4*‐*tai4* effector‐immunity gene pair is not associated with a *tssI* gene cluster.

The T6SS‐1 cluster in *B. cenocepacia* encodes no obvious T6SS‐dependent effectors. However, an earlier bioinformatics survey of the *B. cenocepacia* K56‐2 genome identified ten TssI proteins encoded at other locations within the genome, of which two (BCAL1359 and BCAS0667) contain C‐terminal effector domains (Aubert et al., 2015). Here, using what is known from previously characterized T6SS‐dependent effectors, coupled with bioinformatic tools, we have identified additional putative T6SS‐dependent non‐TssI effectors and their cognate immunity proteins encoded by the *B. cenocepacia *genome. As T6SS‐dependent effector genes in other species are often located within close proximity to *tssI *genes (Lien & Lai, 2017), we used the predicted amino acid sequences of protein products encoded within close proximity to the ten intact *tssI *genes and one disrupted *tssI* gene (BCAL2503) present within the *B. cenocepacia *J2315 genome as queries in BLASTP searches to identify putative functional domains and homology to proteins belonging to established T6SS effector superfamilies (Appendix Figure A6). Twelve putative T6SS effectors were identified using this approach, with each of the *tssI *clusters in *B. cenocepacia *J2315 encoding at least one putative effector. Of the putative effectors identified, six were predicted to be phospholipases (encoded by BCAL1296, BCAL1358, BCAL1366, BCAL2277, BCAM0046, and BCAM0149), five of which belong to the Tle antibacterial effector superfamily (Russell et al., 2013). Of the six remaining effectors, one is a predicted peptidoglycan hydrolase (BCAL1166), two were identified as putative nuclease effectors (BCAL1298 and BCAS0663), of which the latter contains RHS repeats, an additional RHS repeat protein (BCAM2253) containing a RES‐type NAD^+^ glycohydrolase CTD (Skjerning, Senissar, Winther, Gerdes, & Brodersen, 2018), and two homologues of the antibacterial pore‐forming toxin Tse4 (BCAL1292 and BCAL2505; Whitney et al., 2014; LaCourse et al., 2018) were also identified. An additional putative T6SS effector was identified by using homologues of the Tae peptidoglycan hydrolase T6SS effector superfamilies as queries to search the entire translated genome of *B. cenocepacia*, resulting in identification of a Tae4‐Tai4 effector immunity pair (BCAM1464‐BCAM1465) located away from a *tssI* gene cluster. Further details of the putative T6SS effectors identified in these searches are included in Table A6 in Appendix 1, which includes the specific domains identified and putative immunity proteins. It should be noted that the previously described TecA effector (Aubert et al., 2016) is not encoded within a *tssI* gene cluster and was not independently identified in our analysis.

## 4 DISCUSSION

Although some species of bacteria, such as *S. marcescens* and *V. cholerae *V52, do exhibit high basal levels of T6SS activity during growth in laboratory media (Gerc et al., 2015; MacIntyre et al., 2010), in other cases the T6SS is observed to be inactive (Burtnick et al., 2011; Mougous et al., 2006; Zheng, Shin, Cameron, & Mekalanos, 2010), necessitating the use of bacterial strains that have a constitutively active T6SS in order to investigate the functional role of the system and aid in the identification of T6SS‐dependent substrates (Hood et al., 2010; Russell et al., 2011). Here, we demonstrate that the T6SS‐1 of *B. cenocepacia* is active under standard laboratory conditions with sufficient basal activity to allow detection of TssD in concentrated culture supernatant by immunoblotting. This observation is consistent with a previous proteomic study in which a protein identified as “hemolysin‐coregulated protein” (i.e., Hcp or TssD) was detected in the extracellular fraction of strain H111 through 2‐DE coupled mass spectrometry which was not recognized as a T6SS subunit at the time (Riedel, Carranza, Gehrig, Potthast, & Eberl, 2006). These results are also consistent with an investigation that demonstrated TssD secretion in strain K56‐2 could be increased upon inactivation of a global virulence regulator, *atsR* (Aubert et al., 2008). This study showed the presence of very small amounts of a protein corresponding in size to TssD in wild‐type K56‐2 culture supernatants by SDS‐PAGE, which was confirmed by mass spectrometry rather than immunoblotting, as in our study. Moreover, the low abundance of this protein in the secreted fraction led the authors to consider the T6SS activity to be insufficient to use the wild‐type strain in further investigations into the role of the T6SS in *B. cenocepacia*. It is possible that our method of sample preparation and detection in wild‐type K56‐2 was more sensitive than that used in the Aubert and co‐workers study.

The role of the T6SS in interspecies and intraspecies bacterial competition has been recognized as a prominent feature of the system in a variety of T6SS‐containing bacteria, including *P. aeruginosa*, *V. cholerae*, and *S. marcescens* (Hood et al., 2010; MacIntyre et al., 2010; Murdoch et al., 2011). In this study, we provide evidence to support a role for the *B. cenocepacia* T6SS‐1 in competition against two bacterial species, *P. putida* and *E. coli*. We have also identified an arsenal of potential antibacterial cargo effectors that could be delivered by T6SS‐1, notably including peptidoglycan hydrolases. The additional T6SS cluster we identified in *B. cenocepacia* H111 (T6SS‐7) is very unlikely to function in bacterial competition under the conditions tested, as the bacterial competition experiments performed in this study indicated that the level of prey survival was the same in the presence of a mutant attacker with an inactive T6SS‐1 as it was when there was no *B. cenocepacia *attacker strain present (Figure 4a). Our results are consistent with observations in other species that encode a *Burkholderia* T6SS‐1‐type secretion system. This includes the T6SS‐1 in *B. thailandensis*, which was found to be the sole T6SS cluster involved in bacterial competition (Schwarz et al., 2010), and the T6SS‐1 homologue in *Acinetobacter* spp. that was shown to contribute to interbacterial competition (Basler, Ho, & Mekalanos, 2013; Carruthers et al., 2013; Repizo et al., 2015; Weber et al., 2016). They are also consistent with recent observations in the related *Paraburkholderia* species *P. phymatum,* where two non‐T6SS‐1‐type secretion systems (T6SS‐3 and T6SS‐b (T6SS‐8)) were found to be responsible for interbacterial competition against β‐rhizobia strains in vitro and as a consequence were less efficient in root nodulation (de Campos, Lardi, Gandolfi, Eberl, & Pessi, 2017).

The H1‐T6SS in *P. aeruginosa* PAO1 is thought to be triggered by attacks from the T6SS (or T4SS) of neighboring cells as a defensive strategy (Basler & Mekalanos, 2012; Basler et al., 2013; Ho, Basler, & Mekalanos, 2013). As a result, *P. aeruginosa *does not display a fitness advantage over T6SS‐deficient competing species (Basler et al., 2013). The T6SSs in *S. marcescens* Db10 and *V. cholerae* V52, on the other hand, fire indiscriminately and do not require activation from a neighboring attacking bacterium, and thereby confer a fitness advantage on the host bacterium against various Gram‐negative competitor species, such as a *E. coli, Salmonella typhimurium*, *and Pseudomonas fluorescens *(Gerc et al., 2015; MacIntyre et al., 2010). Here, we demonstrate that the T6SS‐1 confers a fitness advantage on *B. cenocepacia* over both T6SS‐positive (*P. putida *KT2440) and T6SS‐negative (*E. coli *SM10(λpir)) bacterial species. This may suggest that, like *S. marcescens* and *V. cholerae*, the T6SS‐1 in *B. cenocepacia* is constitutively active and its activation is not stimulated by external T6SS attacks, which is also supported by the evidence of T6SS activity in wild‐type strains of *B. cenocepacia* H111 and K56‐2 under standard laboratory conditions. This would provide additional support for the idea that the defensive regulatory strategy used by *P. aeruginosa* is atypical among T6SSs (Gerc et al., 2015). In addition, one of the T6SSs in *P. putida* KT2440 has been shown to be highly efficient at killing phytopathogens such as *X. campestris* and *P. syringae* (Bernal, Allsopp, Filloux, & Llamas, 2017). However, our results indicate that *B. cenocepacia *survival is unaffected by the presence of *P. putida. *The constitutive activity of the T6SS‐1 we have observed in *B. cenocepacia *may account for this, where *B. cenocepacia* may be able to subvert *P. putida* before *P. putida *can attack with its own T6SS. Alternatively, *B. cenocepacia *may be immune to the T6SS activity of *P. putida* due to the presence of T6SS immunity proteins with interspecies reactivity, as seen for some Tae‐Tai and Tse‐Tsi effectors–immunity pairs in other species (Russell et al., 2012).

In comparison with the antibacterial T6SSs of other species, the fitness advantage of *B. cenocepacia *over the prey species tested is notably less than that observed in several other attacker species, including *P. aeruginosa, V. cholerae*, *and S. marcescens.* In these species, an active T6SS is responsible for 1,000‐ to 100,000‐fold reduction in the number of recovered prey bacteria in a bacterial competition assay (Hood et al., 2010; MacIntyre et al., 2010; Murdoch et al., 2011), whereas we only observed a modest 10‐ to 58‐fold reduction. This observation may be due to several reasons. For example, T6SS expression and activity may be lower in *B. cenocepacia* than these other T6SS‐positive strains, the prey strains used in our competition assay may produce their own antibacterial factors (such as bacteriocins, siderophores, or effectors secreted by other systems), or there may be inherent differences in growth rates between *B. cenocepacia* and the prey species. However, upon enumerating the *B. cenocepacia *attacker species in our bacterial competition assays, we found that *B. cenocepacia* survival was not affected by coculture with the prey species (Appendix Figure A5). It is also conceivable that the prey used here may have immunity toward specific T6SS effectors due to cross‐reacting T6SS‐immunity proteins between species (Russell et al., 2012). It is possible that by screening a larger panel of bacterial species, a species may be identified that is more susceptible to the T6SS‐1‐dependent antibacterial activity of *B. cenocepacia*. Moreover, as the T6SS‐1 cluster harbors a number of genes that potentially encode post‐translational regulators (i.e., *tagF*, *tagM,* and *tagY*), this system may have the capacity to be further upregulated under certain conditions.

The *B. cenocepacia* T6SS‐1 was first implicated in virulence toward eukaryotes in a signature‐tagged mutagenesis (STM) study carried out in a rat model of chronic lung infection in which transposon insertions within the T6SS‐1 gene cluster were associated with impaired survival of the bacterium (Hunt et al., 2004). In subsequent studies, this group demonstrated that the T6SS‐1 contributes toward cytoskeletal rearrangements and inflammasome activation in *B. cenocepacia*‐infected macrophages through host Rho GTPase inactivation (Aubert et al., 2008; Flannagan et al., 2012; Keith et al., 2009; Rosales‐Reyes et al., 2012; Xu et al., 2014). In contrast to the reported impaired survival of T6SS mutants during rat lung infection (Hunt et al., 2004), more recent evidence suggests that the T6SS may contribute to a pyrin inflammasome‐dependent innate immune response that promotes lung tissue inflammation and bacterial clearance in a mouse infection model (Aubert et al., 2016; Xu et al., 2014). The study by Aubert and co‐workers presented data to show that a putative T6SS effector was responsible for this mechanism.

We have tested several T6SS‐inactive strains of *B. cenocepacia* in three eukaryotic host–pathogen models, nematodes, larvae of the wax worm, and zebrafish larvae (Seed & Dennis, 2008; Uehlinger et al., 2009; Vergunst et al., 2010). We found no significant difference in host survival rates in comparison with infection with the WT strain in any of these infection models, suggesting that the T6SS‐1 in *B. cenocepacia* does not have a functional role in pathogenicity. Of note, we have performed our assays in the presence of a functional *atsR*, so the T6SS is not constitutively upregulated as occurs in the absence of AtsR, and instead activation above basal levels would depend on the presence of the appropriate stimulus (as yet unknown) in any of the model systems. Therefore, the T6SS is either not expressed in these models in the presence of AtsR, or does not contribute to a significant host‐induced protective immune response, as seen in mice (Aubert et al., 2016; Xu et al., 2014). We cannot exclude, however, that in the absence of *atsR*, a measurable effect on virulence could be detected.

To conclude, we have carried out a bioinformatic and functional analysis of the T6SS‐1 in the Bcc species *B. cenocepacia*. We have shown that it is encoded on the large chromosome in nearly all *Burkholderia* species, unlike the other T6SSs associated with members of this genus, which are not conserved in all species and are usually specified by chromosome 2. Therefore, T6SS‐1 can be considered as the ancestral *Burkholderia* T6SS and may serve as a marker for this genus. We also showed that T6SS‐1 was constitutively active in two representative clinical strains and could be used to compete against other bacterial species, including *P. putida *and *E. coli*. This is the first demonstration that T6SS‐1 in a Bcc member plays a role in interbacterial competition and adds to the catalogue of Gram‐negative bacteria that use the T6SS for this purpose. The natural reservoir of *B. cenocepacia *is within the environment, particularly in the soil around plant root systems where many other bacteria compete to establish themselves. It is therefore unsurprising that *B. cenocepacia *has evolved a mechanism for competitive fitness against other bacteria, in a similar manner to other ubiquitous *Burkholderia* and *Paraburkholderia* species (de Campos et al., 2017; Schwarz et al., 2010). Future work will look to identify and characterize the secreted components responsible for the T6SS‐dependent antibacterial activity of *B. cenocepacia*.

## CONFLICT OF INTEREST

The authors declare no conflict of interest.

## AUTHORS CONTRIBUTION

H.L.S., M.S.T., A.C.V., and L.E. conceived and designed experiments, and contributed to the writing of the manuscript. H.L.S., S.S., L.Z., and S.Sch. conducted experiments.

## 5 ETHICS STATEMENT

Protocols and procedures employed in this investigation were reviewed and approved by the appropriate institutional review committees. Zebrafish (*Danio rerio*) were kept and handled in compliance with the guidelines of the European Union for handling laboratory animals (http://ec.europa.eu/environment/chemicals/lab_animals/home_en.htm). Studies performed at VBMI are approved by the Direction Départementale de la Protection des Populations (DDPP) du Gard (ID 30–189–4). Infection experiments were terminated before the larvae reached the free feeding stage and did not classify as animal experiments according to the 2010/63/EU Directive. Care and maintenance of zebrafish was as described previously (Vergunst et al., 2010).

## Data Availability

All data are provided in full in the results section of this paper apart from the DNA sequence contig encompassing *B. cenocepacia* Pc715j T6SS‐1 gene cluster which is available at www.ncbi.nlm.nih.gov/genbank/ under accession number MK051000.

## APPENDIX 1

**Table:** Table A1. Bacterial strains and plasmids used in this study

Strain or plasmid	Genotype or description	Source or reference
***Escherichia coli*** **strains**		
JM83	F^−^ *ara* Δ(l*ac‐proAB*) *rpsL* ϕ80d*lacZ*ΔM15 (Sm^R^)	(Yanisch‐Perron et al., 1985)
SM10(λpir)	*thi‐1 thr leu tonA lacY supE recA*::RP4‐2‐Tc::Mu (Km^R^) (λpir)	(Simon et al., 1983)
S17‐1(λpir)	*thi proA hsdR recA* RP4‐2‐*tet*::Mu‐1 *kan*::Tn*7* integrant (Tp^R^, Sm^R^) (λpir)	(Simon et al., 1983)
CC118(λpir)	*araD139* Δ(*ara‐leu*)*7697* Δ*lacX74 galE galK phoA20 thi*‐*1 rpsE argE*_(am)_ *recA1* λpir *rpoB* (Rf^R^)	(Herrero et al., 1990)
OP50	*E. coli* B uracil auxotroph; food source for *C. elegans*	(Brenner, 1974)
***Pseudomonas putida*** **strains**		
KT2440	Spontaneous r^‐^ derivative of mt‐2	(Bagdasarian et al., 1981)
***Burkholderia cenocepacia*** **strains**		
H111	CF isolate	(Römling et al., 1994)
K56‐2	CF isolate, ET12 lineage	(Mahenthiralingam et al., 2000)
Pc715j	CF sputum isolate, ET12 lineage	(McKevitt et al., 1989; Darling et al., 1998)
H111‐*tssA*::Tp	H111 with *tssA* disrupted by *dfrB2* cassette (Tp^R^)	(Dix et al., 2018)
H111‐*tssK*::Tp	H111 with *tssK* disrupted by *dfrB2* cassette (Tp^R^)	This study
H111‐*tssM*::Tp	H111 with *tssM* disrupted by *dfrB2* cassette (Tp^R^)	This study
H111‐Δ*tssM*	H111 with an in‐frame deletion of the internal *Xho*I fragment of *tssM*	(Dix et al., 2018)
H111‐*tagY*::Tp	H111 with *tagY* disrupted by *dfrB2* cassette (Tp^R^)	This study
K56‐2*‐tssA*::Tp	K56‐2 with *tssA* disrupted by *dfrB2* cassette (Tp^R^)	This study
K56‐2‐*tssM*::Tp	K56‐2 with *tssM* disrupted by *dfrB2* cassette (Tp^R^)	This study
Pc715j‐*tssA*::Tp	Pc715j with *tssA* disrupted by *dfrB2* cassette (Tp^R^)	This study
Pc715j‐*tssK*::Tp	Pc715j with *tssK* disrupted by *dfrB2* cassette (Tp^R^)	This study
Pc715j‐*tssM*::Tp	Pc715j with *tssM* disrupted by *dfrB2* cassette (Tp^R^)	This study
***Plasmids***		
pBBR1MCS	Mobilizable BHR cloning vector, pBBR1‐replicon (Cm^R^)	(Kovach et al., 1994)
pBluescriptII KS (+)	General cloning vector, ColE1‐derived phagemid, *lacZ*α MCS (Ap^R^)	(Alting‐Mees and Short, 1989)
p34E‐Tp	p34E containing *dfrB2* gene (Ap^R^, Tp^R^)	(DeShazer and Woods, 1996)
pSHAFT2	Suicide vector, R6K‐derived replicon, *ori*T^+^ (Ap^R^, Cm^R^)	(Shastri et al., 2017)
pBBR1‐*tssK*	pBBR1MCS containing *tssK* from *B. cenocepacia* H111 cloned between *Hin*dIII and *Bam*HI (Cm^R^)	This study
pBBR1‐*tssM’*	pBBR1MCS‐1 containing 1.2 kbp N‐terminal fragment of *tssM* from *B. cenocepacia* H111 cloned between *Xba*I and *Xho*I (Cm^R^)	This study
pBBR1‐*tssM(+)*	pBBR1MCS containing full‐length *tssM* from *B. cenocepacia* H111 cloned between *Acc*65I and *Xba*I (Cm^R^)	This study
pBluescriptII‐*tagY*	pBluescriptII containing *tagY* from *B. cenocepacia* H111 cloned between *Bam*HI and *Xho*I sites (Ap^R^)	This study
pBBR1‐*tssK*::Tp	pBBR1MCS containing *tssK* disrupted by *dfrB2* cassette at *Eco*RI site in same orientation as *tssK* (Cm^R^, Tp^R^)	This study
pBBR1‐*tssM’*::Tp	pBBR1MCS containing *tssM’* disrupted by *dfrB2* cassette at *Bam*HI site in reverse orientation as *tssM’* (Cm^R^, Tp^R^)	This study
pBluescriptII‐*tagY*::Tp	pBluescriptII containing *tagY* disrupted by the *dfrB2* cassette at *Zra*I site in the same orientation as *tagY* (Ap^R^)	This study
pSHAFT2‐*tssA*::Tp	pSHAFT2 containing *tssA*::Tp allele from pBBR1‐*tssA*::Tp cloned between *Xho*I and *Not*I (Ap^R^, Cm^R^, Tp^R^)	(Dix et al., 2018)
pSHAFT2‐*tssK*::Tp	pSHAFT2 containing *tssK*::Tp allele from pBBR1‐*tssK*::Tp cloned between *Xho*I and *Not*I (Ap^R^, Cm^R^, Tp^R^)	This study
pSHAFT2‐*tssM’*::Tp	pSHAFT2 containing *tssM’*::Tp allele from pBBR1‐*tssM’*::Tp cloned between *Xho*I and *Not*I (Ap^R^, Cm^R^, Tp^R^)	This study
pSHAFT2‐*tagY*::Tp	pSHAFT2 containing *tagY*::Tp allele from pBluescriptII‐*tagY*::Tp cloned between *Sal*I and *Xba*I (Ap^R^, Cm^R^, Tp^R^)	This study

**Abbreviations:** Ap^R^, ampicillin‐resistant; Cm^R^, chloramphenicol‐resistant; Km^R^, kanamycin‐resistant; Rf^R^, rifampicin‐resistant; Sm^R^, streptomycin‐resistant; Tc^R^, tetracycline‐resistant; Tp^R^, trimethoprim‐resistant; BHR, broad host range.

**Table:** Table A2. Primers used in this study

Primer ID	Primer sequence^a^
iotAfor	5’‐GCGCAAGCTTCACGCGACATCTCATGCATC
iotArev2	5’‐ATCACGAAGAGCATTCCGCC
tssKfor	5’‐GCGCAAGCTTCCACATTAACCGGATTTGAC
tssKrev	5’‐GCGCGGATCCTCATGATGTGACCGCGATCA
tssK‐OPfor	5’‐GCGATTTAATTCGGGCACGA
tssK‐OPrev	5’‐ACAGCAAATCGAGCAGCGAA
tssMfor	5’‐GCGCTCTAGAGGAACCTGAACGTCCTATGC
tssMrev	5’‐GCGCCTCGAGCTGTTGGTTTCGCCTTCCTG
tssMforAcc65I	5’ ‐GCGCGGTACCTTAAAATCGCACCGGAACCTGAAC
tssMrevXbaI	5’‐GCGCTCTAGAATTTGCGCCTGTACGGTTTG
tssM‐OPfor	5’‐TCATCCCGTTTGACAGCATG
tssM‐OPrev	5’‐AGAAGCCGTTCTTCGAGAAC
tagYfor	5’‐GCGCCTCGAGTAAAGTGCGCCGGAAAATTCAAA
tagYrev	5’‐GCGCGGATCCCAGTGTCACGCGACATCATA
tagY‐OPfor	5’‐GCGCTCTAGAATCCCCGGAAATTGGAATTG
tagY‐OPrev	5’‐GCGCAAGCTTGGTAAGGAAAGGAGACGTAT

^a^Sequences specifying restriction endonuclease cleavage sites are underlined.

**Table:** Table A3. T6SS‐1 gene loci in the *Burkholderia* genus

Species	Strain	Chr^b^	Locus^a^	Old locus/alias^a^
***Burkholderia cepacia*** **complex**				
*B. ambifaria*	AMMD	1	BAMB_RS01920‐BAMB_RS02010	Bamb_0377‐Bamb_0395
*B. anthina*	AZ‐4‐2‐10‐S1‐D7	1	WS64_RS00645‐WS64_RS00755	WS64_00645‐WS64_00755
*B. catarinensis*	89	n/a	BFF94_26275‐BFF94_35355	n/a
*B. cenocepacia*	J2315	1	QU43_RS38220‐QU43_RS38300	BCAL0337‐BCAL0353
*B. cepacia*	UCB 717	1	APZ15_RS05925‐APZ15_RS06005	APZ15_05925‐APZ15_06005
*B. contaminans*	170816	1	C3743_RS13835‐ C3743_RS13945	C3743_28295‐C3743_28405
*B. diffusa*	RF2‐non‐BP9	1	WI26_RS01890‐WI26_RS01990	WI26_01890‐WI26_01990
*B. dolosa*	AU0158	1	AK34_RS26290‐AK34_RS26210	AK34_2669‐AK34_2653
*B. lata*	383	1	BCEP18194_RS07870‐BCEP18194_RS07970	Bcep18194_A3555‐Bcep18194_A3577
*B. latens*	AU17928	1	WK25_RS00385‐WK25_RS00305	WK25_00385‐WK25_00305
*B. metallica*	FL‐6‐5‐30‐S1‐D7	1	WJ16_RS01935‐WJ16_RS02050	WJ16_01935‐WJ16_02050
*B. multivorans*	ATCC 17616	1	BMULJ_RS01495‐BMULJ_RS01645	BMULJ_00300‐BMULJ_00329
*B. paludis*	MSh1	n/a	GQ56_0123510‐GQ56_0123430	n/a
*B. pseudomultivorans*	SUB‐INT23‐BP2	1	WS57_RS19835‐WS57_RS19915	WS57_19795‐WS57_19875
*B. puraquae*	CAMPA 1040	n/a	B7G54_RS33210‐B7G54_RS33130	B7G54_33195‐B7G54_33115
*B. pyrrocinia*	DSM 10685	1	ABD05_RS07950‐ABD05_RS08030	ABD05_07950‐ABD05_08030
*B. seminalis*	FL‐5‐4‐10‐S1‐D7	1	WJ12_RS01985‐WJ12_RS02085	WJ12_01985‐WJ12_02085
*B. stabilis*	ATCC BAA‐67	1	BBJ41_RS12095‐BBJ41_RS12210	BBJ41_12095‐BBJ41_12210
*B. stagnalis*	MSMB735	1	WT74_RS02265‐WT74_RS02350	WT74_02260‐WT74_02345
*B. territorii*	RF8‐non‐BP5	1	WS51_RS12720‐WS51_RS12820	WS51_12715‐WS51_12815
*B. ubonensis*	MSMB22	1	BW23_RS21305‐BW23_RS21205	BW23_1274‐BW23_1254
*B. vietnamiensis*	G4	1	BCEP1808_RS02265‐BCEP1808_RS02350	Bcep1808_0456‐Bcep1808_0473
***Burkholderia pseudomallei*** ** group**				
*B. humptydooensis*	MSMB122	1	WS76_02215‐WS76_02295	n/a
*B. mallei* ^c^	NCTC 10229	1	BMA10229_RS17595‐BMA10229_RS17645	BMA10229_A1710‐BMA10229_A1720
*B. oklahomensis*	EO147	1	DM82_RS14115‐DM82_RS14035	DM82_2790‐DM82_2774
*B. pseudomallei*	K96243	1	BPSL3111‐BPSL3095	AQ15_RS22375‐AQ15_RS22455
*B. thailandensis*	E254	1	BTH_RS27330‐BTH_RS27250	BTH_I2968‐BTH_I2951
*B. singularis*	LMG 28154	‐	‐	
**Phytopathogens**				
*B. gladioli*	ATCC 10248	1	BM43_RS25670‐BM43_RS25750	BM43_1793‐BM43_1809
*B. glumae*	BGR1	1	BGLU_RS01925‐BGLU_RS02005	bglu_1g03850‐bglu_1g04010
*B. plantarii*	ATCC 43733	1	bpln_RS01775‐bpln_RS01855	bpln_1g03440‐bpln_1g03600

**Abbreviations:** Chr, chromosome; n/a, not applicable; ‐, not present.

^a^Gene loci refer to the first (*tssL*) and last (*tagY*) genes in the T6SS‐1 gene cluster in these species as shown in Figure 1.

^b^If n/a is stated, chromosome location was not available as the loci coordinates were obtained from draft assemblies consisting of contigs.

^c^Truncated cluster that lacks *tssL‐tssD*.

**Table:** Table A4. *Paraburkholderia* species containing homologous loci of the *Burkholderia* T6SS‐1 cluster identified through bioinformatics analysis

Species	Strain	Chr^b^	Locus^a^	Old locus/alias^a^
*P. acidipaludis*	NBRC 101816	n/a	BAC01S_RS24625‐BAC01S_RS24720	n/a
*P. aspalathi*	LMG 27731	n/a	BM438_RS23205‐BM438_RS23285	SAMN05192563_101653‐SAMN05192563_101669
*P. bannensis*	NBRC 103871	n/a	BBA01S_RS03935‐BBA01S_RS03850	n/a
*P. bryophila*	376MFSha3.1	n/a	H281_RS0127575‐H281_RS0127655	n/a
*P. caledonica*	NBRC 102488	n/a	BCA01S_RS25625‐BCA01S_RS25545	n/a
*P. caribensis*	DSM 13236	3	C2L66_RS31465‐C2L66_RS31545	C2L66_31465‐C2L66_31545
*P. caryophylli*	Ballard 720	n/a	C0Z17_RS09175‐C0Z17_RS09265	C0Z17_09180‐C0Z17_09270
*P. dilworthii*	WSM3556	n/a	F759_RS0111885‐F759_RS0111970	n/a
*P. eburnea*	LMG 29537	n/a	BX588_RS11555‐BX588_RS30870	BX588_10514‐BX588_1421
*P. fungorum*	GAS106B	n/a	BLS41_RS32405‐BLS41_RS32325	SAMN05443245_6595‐SAMN05443245_6579
*P. ginsengiterrae*	DCY85	n/a	A6V36_RS34655‐A6V36_RS34735	A6V36_13555‐A6V36_13635
*P. graminis*	C4D1M	n/a	BGRAMDRAFT_RS31300‐BGRAMDRAFT_RS31220	BgramDRAFT_6363‐BgramDRAFT_6347
*P. insulsa*	LMG 28183	n/a	BX589_RS19295‐BX589_RS19215	BX589_111106‐BX589_11190
*P. kururiensis*	M130	n/a	G118_RS0127045‐G118_RS0127125	n/a
*P. megapolitana*	LMG 23650	n/a	BM166_RS27120‐BM166_RS27200	SAMN05192543_109158‐SAMN05192543_109174
*P. nodosa*	CNPSo 1341	n/a	BFD71_RS23080‐BFD71_RS22965	n/a
*P. oxyphila*	NBRC 105797	n/a	BO1_RS31110‐BO1_RS23945	n/a
*P. phenazinium*	GAS95	n/a	BUS12_RS10920‐BUS12_RS10830	SAMN05444165_2229‐SAMN05444165_2211
*P. phenoliruptrix*	BR3459a	2	BUPH_RS28500‐BUPH_RS28580	BUPH_06127‐BUPH_06111
*P. phytofirmans*	PsJN	2	BPHYT_RS24375‐BPHYT_RS24455	Bphyt_4909‐Bphyt_4925
*P. rhynchosiae*	WSM 3937	n/a	C0Z16_RS18310‐C0Z16_RS18230	C0Z16_18290‐C0Z16_18210
*P. sediminicola*	LMG 24238	n/a	BLT79_RS22400‐BLT79_RS22480	SAMN05192547_102957‐SAMN05192547_102973
*P. soli*	GP25‐8	n/a	C0Z19_RS08700‐C0Z19_RS08620	C0Z19_08720‐C0Z19_08640
*Paraburkholderia sp*.	BL18I3N2	n/a	B0G75_RS24645‐B0G75_RS24565	B0G75_110100‐B0G75_11084
*Paraburkholderia sp*.	BL21I4N1	n/a	B0G83_RS22810‐B0G83_RS22905	B0G83_109119‐B0G83_109138
*Paraburkholderia sp*.	BL25I1N1	n/a	B0G73_RS11290‐B0G73_RS11370	B0G73_106134‐B0G73_106151
*Paraburkholderia sp*.	C35	n/a	DK391_RS17735‐DK391_RS17655	n/a
*Paraburkholderia sp*.	GV068	n/a	C8K18_RS26440‐C8K18_RS26520	C8K18_115102‐C8K18_115118
*Paraburkholderia sp*.	GV072	n/a	C8K19_RS26210‐C8K19_RS26130	C8K19_11551‐C8K19_11535
*P. symbiotica*	JPY 581	n/a	C0Z20_RS18355‐C0Z20_RS18280	C0Z20_18350‐C0Z20_18275
*P. terricola*	LMG 20594	n/a	BUE39_RS22490‐BUE39_RS22570	SAMN05192548_102957‐SAMN05192548_102973
*P. tropica*	LMG 22274	n/a	BMY06_RS23990‐BMY06_RS23890	SAMN05216550_114137‐SAMN05216550_114117

**Abbreviations:** Chr, chromosome; n/a, not applicable.

^a^Gene loci refer to the first (*tssL*) and last (*tagY*) genes in the T6SS‐1 gene cluster in these species as shown in Figure 1. *P. acidipaludis* is an exception to this, where the gene loci refer to *tssL* to *tagN*.

^b^If n/a is stated, chromosome location was not available as the loci coordinates were obtained from draft assemblies consisting of contigs.

**Table:** Table A5. Strain‐specific T6SS‐7 gene loci in *Burkholderia* and related species

Species	Strain	Chr^b^	Locus^a^	Old locus/alias^a^
***Burkholderia***				
*B. cenocepacia*	H111	2	I25_RS17325‐I35_RS17415	I35_0565‐I35_0547
	DWS 37E‐2	2	DM40_RS13140‐DM40_RS13060	DM40_4776‐DM40_4759
	FL‐5‐3‐30‐S1‐D7	2	WJ11_00625‐WJ11_00725	n/a
	VC12308	2	A8E75_RS00805‐A8E75_RS00910	A8E75_00815‐A8E75_00910
	D2AES	n/a	W5I_RS0113450‐W5I_RS0113540	n/a
	PC184 Mulks	1^c^	B9Z07_RS01130‐B9Z07_RS01225	B9Z07_01130‐B9Z07_01230
	TAtl‐371	n/a	BLS50_RS24480‐BLS50_RS24390	SAMN05443026_4796‐SAMN05443026_4778
*B. ambifaria*	RZ2MS16	n/a	AS146_RS14130‐AS146_RS14210	n/a
*B. cepacia*	LK29 14	n/a	VL15_RS08940‐VL15_RS08850	VL15_08935‐VL15_08845
*B. diffusa*	MSMB0010	n/a	WJ30_RS22370‐WJ30_RS22295	WJ30_23825‐WJ30_23750
*B. dolosa*	AU0158	2	AK34_RS03975‐AK34_RS04060	AK34_3963‐AK34_3981
*B. latens*	AU17928	2	WK25_RS26375‐WK25_RS26295	WK25_26365‐WK25_26285
*B. metallica*	FL‐6‐5‐30‐S1‐D7	2	WJ16_RS22925‐WJ16_RS23015	WJ16_22900‐WJ16_22990
*B. pseudomultivorans*	MSMB574	n/a	WT57_RS16705‐WT57_RS16620	WT57_21995‐WT57_21910
*B. puraquae*	CAMPA 1040	n/a	B7G54_RS20355‐B7G54_RS20270	B7G54_20345‐B7G54_20260
*B. pyrrocinia*	MSMB1755	n/a	WJ63_RS05815‐WJ63_RS05755	WJ63_27610‐WJ63_27550
*B. stabilis*	LA20W	n/a	BSLA_02f3182‐BSLA_02r3154	n/a
*B. stagnalis*	MSMB1147	n/a	WT05_RS32155‐WT05_RS32230	WT05_32120‐WT05_32195
*B. territorii*	MSMB1917	n/a	WT40_RS07200‐WT40_RS07125	WT40_07845‐WT40_07770
*B. ubonesis*	MSMB2006	n/a	WK05_RS31815‐WK05_RS31895	WK05_23690‐WK05_23770
*B. vietnamiensis*	FL‐2‐2‐30‐S1‐D0	n/a	WJ01_RS25680‐WJ01_RS25595	WJ01_26115‐WJ01_26030
*B. glumae*	BGR1	2	BGLU_RS23295‐BGLU_RS23205	bglu_2g11110‐bglu_2g10910
**Species related to ** ***Burkholderia***				
*P. tuberum*	DUS833	n/a	BLU10_RS24530‐BLU10_RS24630	SAMN05445850_5072‐SAMN05445850_5093
*C. metallidurans*	CH34	n/a	RMET_RS03120‐RMET_RS03215	Rmet_0617‐Rmet_0637

**Abbreviations:** Chr, chromosome; n/a, not applicable.

^a^Gene loci refer to the first (*tssE*) and last (*tssJ*) genes in the T6SS‐7 gene cluster in these species as shown in Figure 2.

^b^If n/a is stated, chromosome location was not available as the loci coordinates were obtained from draft assemblies consisting of contigs.

^c^Chromosome designation may be incorrect for this genome assembly as the largest chromosome has been designated chromosome 3 and second largest chromosome 1. Furthermore, the nucleotide sequence of chromosome 2 of PC184 Mulks shares significant sequence homology with chromosome 1 of *B. cenocepacia* J2315 and H111, indicating that chromosome 3 should be designated as chromosome 2, which would mean the T6SS‐7 cluster is located on chromosome 2 in this strain, as observed in other *B. cenocepacia* strains.

**Table:** Table A6. Putative T6SS‐dependent effectors localised to *tssI* gene clusters in *B. cenocepacia* J2315 identified *in silico*

Effector locus^a^	Chr	*tssI* locus^a^	Predicted activity	Functional domain/homology^b^	Immunity locus^a^
BCAL1166 (QU43_RS42350)	1	BCAL1165 (QU43_RS42345)	Peptidoglycan hydrolase	M23 peptidase (PF01551), SH3_3 (PF08239), Lysozyme‐like CTD (cl00222)	BCAL1167 (QU43_RS73515)
BCAL1292 (QU43_RS42955)	1	BCAL1294 (QU43_RS42965)	Pore‐forming	Tse4	BCAL1291 (QU43_RS42950)
BCAL1296 (QU43_RS42975)	1	BCAL1294 (QU43_RS42965)	Phospholipase	PAAR_like/DUF4150 (PF13665), Lipase_3 (cd00519)	BCAL1297 (QU43_RS42980)
BCAL1298 (QU43_RS75480)	1	BCAL1294 (QU43_RS42965)	Nuclease	GH‐E HNH/ENDO VII nuclease (PF14410)	BCAL1299 (QU43_RS42990)
BCAL1358 (QU43_RS43285)	1	BCAL1355 (QU43_RS43270)	Phospholipase	Tle1/DUF2235 (PF09994)	BCAL1357 (QU43_RS43280)
BCAL1366 (QU43_RS43320)	1	BCAL1362 (QU43_RS43300)	Phospholipase	Tle1/DUF2235 (PF09994)	BCAL1365 (QU43_RS43315)
BCAL2277 (QU43_RS48015)	1	BCAL2279 (QU43_RS48025)	Phospholipase	Tle3, DUF3274 (PF11678)	BCAL2276 (QU43_RS48010), BCAL2274 (QU43_RS48000), BCAL2272 (QU43_RS47990)
BCAL2504 (QU43_RS49175)	1	BCAL2503^c^ (QU43_RS49165)	Pore‐forming	Tse4	BCAL2505 (QU43_RS49180)
BCAM1464 (QU43_RS74705)	2	N/A^d^	Peptidoglycan hydrolase	Tae4	BCAM1465 (QU43_RS61680)
BCAM0046 (QU43_RS54630)	2	BCAM0043 (QU43_RS54615)	Phospholipase	Tle1/DUF2235 (PF09994)	BCAM0045 (QU43_RS54625)
BCAM0149 (QU43_RS55145)	2	BCAM0148 (QU43_RS55140)	Phospholipase	Tle5/PLDc_SF (cl15239)	BCAM0150 (QU43_RS55150), BCAM0152 (QU43_RS55160)
BCAM2253 (QU43_RS65635)	2	BCAM2254 (QU43_RS65645)	NAD^+^ glycohydrolase	PAAR_RHS (cd14742), RhsA (COG3209), RES CTD (smart00953,214933)	QU43_RS75580
BCAM2252 (QU43_RS65630)	2	BCAM2254 (QU43_RS65645)	Unknown	RhsA (COG3209)	BCAM2251a (QU43_RS65625)
BCAS0663 (QU43_RS71975)	3	BCAS0667 (QU43_RS71995)	Nuclease	PAAR_RHS (cd14742), RhsA (COG3209), HNH/ENDO VII superfamily with WHH CTD (PF14414)	BCAS0662 (QU43_RS71970), BCAS0661c (QU43_RS75295)

^a^New/alias locus names given in parentheses.

^b^Pfam or Conserved Domain Database accession numbers of the domains are given in parentheses.

^c^Disrupted *tssI* gene that is very likely to be non‐functional as the encoded product is truncated at the N‐terminus and fused to a *tssF* orthologue.

^d^Effector not located near a *tssI* cluster.

## APPENDIX 2

### Further details regarding construction of *B. cenocepacia* mutants

Primer pairs tssA‐OPfor and tssA‐OPrev, tssK‐OPfor and tssK‐OPrev, tssM‐OPfor and tssM‐OPrev and tagY‐OPfor and tagY‐OPrev were used for PCR screening of candidate *tssA*::Tp, *tssK*::Tp, *tssM*::Tp and *tagY*::Tp mutants, respectively.
